# Structure, Electrochemistry, and Phase Evolution of Al-Substituted Na_2/3_[Ni_1/3‑y_Mn_2/3‑z_Al_*y*+*z*_]O_2_ as a Sodium-Ion Battery Cathode Material

**DOI:** 10.1021/acs.chemmater.5c03184

**Published:** 2026-05-19

**Authors:** Anthony T. Pacileo, Patrick Deegan, Hao Liu

**Affiliations:** † Materials Science and Engineering Program, 14787 Binghamton University, Binghamton, New York 13902, United States; ‡ Department of Chemistry, Binghamton University, Binghamton, New York 13902, United States

## Abstract

Sodium layered transition metal oxide is a promising cathode material for Na-ion batteries. The P2-type structure and especially the Na_2/3_[Ni_1/3_Mn_2/3_]­O_2_ (NaNM) composition are desirable for their rate capability and energy density. Unfortunately, these materials are hindered by their rapid capacity loss during charge/discharge cycling. While prior work has shown the benefits of Al doping in improving its capacity retention, it remains unclear whether and where Al is substituted in the bulk crystalline phase, especially given the honeycomb ordering of Ni and Mn in NaNM, and how Al-substitution affects the structural evolution during electrochemical cycling. In this work we synthesize Al-substituted NaNM via two substitution strategies–aliovalent and “isovalent” substitution. Complementary X-ray powder diffraction (XRD) and neutron powder diffraction (NPD) are used to determine the effects of Al-substitution strategies on the crystal structure and show a preference of Al to substitute Ni, not Mn. Operando XRD is used to track phase evolution of the Al-substituted phases and reveals a reduced lattice mismatch during the high voltage phase transition as Al content increases. This work provides insights into the complex elemental substitution in NaNM and serves as the basis for developing future substitution strategies.

## Graphical abstract

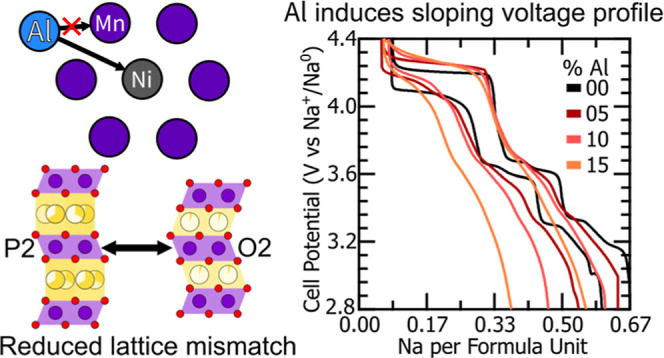

## 1 Introduction

Na-ion batteries (NIB) have been investigated as an alternative to Li-ion batteries (LIB) for electrochemical energy storage. Especially relevant are layered transition metal oxides (LTMOs), which are analogous to the state-of-the-art LIB LTMO cathodes and afford facile intercalation of the Na^+^ ion. One of the widely explored compositions is the P2-type Na_2/3_[Ni_1/3_Mn_2/3_]­O_2_ (NaNM). The naming of this structure as “P2-type” follows the convention from Delmas et al.,^1^ as it contains trigonal prismatic alkali ion sites and has translational symmetry with a 2-layer period along the stacking direction of the transition metal oxide layers. This composition was initially prepared and investigated by Paulsen and Dahn as a precursor for lithium cathode materials.^2^ NaNM has a theoretical capacity of 260 mA h g^–1^ and energy density of >700 W h kg^–1^ (vs Na metal) for the (de)­intercalation of Na^+^ ions in the composition range of 0 < *x* < 1 for Na_*x*_[Ni_1/3_Mn_2/3_]­O_2_ (606 W h kg^–1^ and 170 mA h g^–1^ for 0.03 < *x* < 0.66).^3^ However, this theoretical capacity is not realized in practice because of the rapid capacity fade when cycled at high voltage where *x* < 1/3.^4–8^ Overcoming this deteriorating effect is necessary to enable high-performance cathode materials for NIB.

One of the leading causes of this poor capacity retention is attributed to the high-voltage phase transition at 4.2 V (vs Na^+^/Na^0^), where a two-phase reaction takes place between the P2 and O2 phases accompanied by the nominal Ni^4+^/Ni^3+^ and anionic redox reactions.^9^ The transformation from the P2 to the O2 phase proceeds via the gliding of the transition metal (TM) oxide layers, which leads to a change in the Na coordination environment from trigonal prismatic (as in the P2 phase) to octahedral (as in the O2 phase).^7,9,10^ Intercalation reactions which proceed via a first-order phase transition typically require a larger energy barrier than the solid-solution mechanism.^11^ This first-order phase transition could also account for the larger overpotential observed for the electrochemical cycling in the high-voltage plateau. Another issue derived from this reaction mechanism is stress created at the interface between the O2 and P2 domains, which can cause particle cracking and delamination that compromise the integrity of the electrode particles.^12^ With regards to the chemical nature of the charged phase, activation of the highly oxidized Ni^4+^ and anionic redox causes voltage hysteresis and Coulombic inefficiency.^13^ These materials also suffer from electrochemical side reactions at the particle surface due to the highly oxidizing environment above 4.2 V.^14^ As a result, the NaNM material can only be reversibly cycled between 4.1 and 2.8 V, within the composition range of 1/3 ≤ *x* ≤ 2/3 in Na_*x*_[Ni_1/3_Mn_2/3_]­O_2_.

Elemental substitution at the transition metal site is a popular strategy to improve the performance of NaNM.^7,15–18^ Substitution with Al has been shown to improve the cycling stability: Hasa et al. reported a partial substitution of Ni with Al for Na_0.6_[Ni_0.22_Mn_0.66_Al_0.11_]­O_2_ that maintains the same undistorted P2-type structure and exhibits significantly improved capacity retention;^17^ whereas Kubota et al. reported an Al-substitution in place of both Ni and Mn in Na_2/3_[Ni_11/36_Mn_23/36_Al_2/36_]­O_2_ with a major reduction in voltage polarization and capacity decay.^18^ These studies tend to agree on the mechanism of capacity stabilization: the inactive Al^3+^ ion promotes some Na to remain in the structure at the fully charged state, which acts as a pillar to support the layered structure.^17–19^ However, there is a significant discrepancy in the voltage profile of the Al-substituted materials: Jiang and Kubota showed similar flat plateaus to the unsubstituted,^18,19^ while Hasa showed a highly sloped voltage profile.^17^ This discrepancy implies the different effects of Al-substitution on the underlying phase transition. Hasa also reported a capacity that exceeds the theoretical capacity for Ni^2+^/Ni^4+^ redox, raising the question of the charge compensation mechanism in the Al-substituted phase.^17^ Another key question is whether Al-substitution throughout the lattice occurs since Al can also segregate at the particle surface, and such Al-rich coatings have been reported to benefit the electrochemical performance,^20,21^ which cannot be ruled out without crystallographic evidence of the Al concentration.

Of particular interest to the stoichiometric NaNM phase is that the Ni^2+^ and Mn^4+^ ions form a honeycomb ordering in the transition metal layer, which is typically observed for phases with the general formula Na_*x*_[M′_1/3_M″_2/3_]­O_2_ ^22–26^ and yields two crystallographic sites for potential Al-substitution.^2,27^ This strong transition metal order creates a structured energy landscape in the Na sites which drives Na/vacancy ordering,^28^ shapes diffusion mechanics,^29^ and controls phase transformation behavior.^30,31^ Such distinct sites could lead to preferential occupation by Al, which has not been studied and the effect of which has not been addressed. Lastly, the effect of Al-substitution on the phase evolution remains undetermined. Therefore, to inform better substitution strategies, it is important to elucidate the complex relationship between elemental substitution, crystal structure, phase evolution, and electrochemical behavior.

In this work, we investigate the effect of two Al-substitution strategies–aliovalent vs isovalent substitution–on the crystal structure, phase transition, and electrochemical behavior of P2 NaNM. For the aliovalent substitution scheme, Al was intended to only substitute for the Ni site, leading to the general formula of Na_*x*_[Ni_(1/3)‑*y*_Mn_2/3_Al_*y*_]­O_2._ For the isovalent substitution scheme, Al was intended to equally substitute for both Ni and Mn sites, leading to the general formula of Na_2/3_[Ni_(1/3)‑(*y*/2)_Mn_(2/3)‑(*y*/2)_Al_*y*_]­O_2_. Note that these nominal compositions are only based on the filling ratio of the metal oxide precursors and these are not representative of the composition of the P2 phase. These are referred to with “A” for aliovalent samples and “I” for isovalent samples, followed by their nominal Al stoichiometry (e.g., A02, A05, A07 corresponds to *y* = 0.02, 0.05, and 0.07, respectively, in Na_*x*_[Ni_(1/3)‑*y*_Mn_2/3_Al_*y*_]­O_2_). Combined X-ray and neutron diffraction was employed to determine the crystal structure of samples resulting from both substitution schemes. Operando synchrotron X-ray diffraction was used to track the phase evolution during electrochemical cycling. The electrochemical behavior of the substituted samples was interpreted considering both the structural and phase evolution results.

## 2 Experimental Section

### 2.1 Synthesis

Materials were synthesized using a solid state reaction method. In a typical synthesis 10 g were prepared. Stoichiometric amounts of sodium carbonate (99.95%, Thermo Scientific), nickel­(II) oxide (99%, Sigma), manganese­(III) oxide (99%, Sigma), and γ-alumina (99%, 20 nm aps, Alfa Aesar) were weighed with a digital balance. The stoichiometries of the aliovalent and isovalent Al-substituted samples were controlled by adjusting the ratios of the Ni, Mn, and Al precursors. Sodium carbonate was added at 8 mol % excess to account for evaporation. Precursors were mixed via a high energy ball mill for 15 min and then compressed into pellets at 50 bar for 1 min. The pellets were placed in alumina crucibles with additional precursor powder covering them on all sides. The materials were then heated in air in an MTI KSL-1200X muffle furnace from room temperature to 1000 °C at 5 °C min^–1^ and maintained this temperature for 12 h, then cooled at the natural rate to room temperature which takes roughly 4 h. The products were ground to a fine powder in an agate mortar and pestle. Products were stored in an Ar filled glovebox with water and oxygen contents of <0.5 ppm.

### 2.2 Electrochemistry

The cathode materials were prepared into film electrodes coated on aluminum foils. 80 wt % active material, 10 wt % polyvinylidene fluoride as a binder, and 10 wt % carbon black were mixed with *N*-methylpyrrolidinone in a 1:2.25 ratio of solids to solvent by mass in a Thinky automatic mixer at 2000 rpm for 5 min after adding each component, in the order of solvent and binder, then conductive additive, then active material, for a total of 15 min of mixing. This slurry was applied onto a piece of aluminum foil with a 30 μm doctor blade, then dried at 80 °C in a vacuum oven overnight. These dried electrodes with 1–5 mg cm^–2^ active material loading were punched into 12 mm diameter disks, weighed, and assembled into 2032 size coin cells. The cells used Na metal as an anode, glass fiber separators, and a 1 M sodium hexafluorophosphate in propylene carbonate electrolyte. Galvanostatic cycling was done from 2.8 to 4.3 V vs Na^+^/Na^0^ at a rate of C/10 with C = 173 mA h g^–1^.

### 2.3 Operando X-Ray Diffraction Cells

Customized operando cells were prepared using similar methods to the typical electrochemistry. The electrode slurries were coated with a 200 μm doctor blade to achieve high active mass loadings of 15–20 mg cm^–2^ which are necessary to yield sufficient signal-to-noise for diffraction measurement in transmission geometry. The cases were 2016 size coin cells with rectangular windows, which were sealed by polyimide tape on the interior and epoxy resin on the exterior, as reported previously by our group.^32^ These used Na metal anodes which were spread thin across the anode case to cover the window, ensuring a complete reaction in the X-ray gauge volume. The separator and electrolyte were the same as the typical electrochemistry, with glass fiber separators and a 1 M sodium hexafluorophosphate in propylene carbonate electrolyte. The experiment was arranged with the beam transmitted through the entire cell from the anode to the cathode.

### 2.4 Synchrotron X-Ray Diffraction

Operando XRD was done at the National Synchrotron Light Source II, Brookhaven National Laboratory at beamline 28-ID-2. For this experiment, multiple cells were loaded on a motorized sample stage, which automatically rotated each cell into the beam every 7 min 52 s (0.1312 h). A stationary PerkinElmer 40 × 40 cm detector was placed 1.3 m behind the cells, and with an X-ray wavelength of 0.1811 Å, affording coverage of *d* from 13 to 0.7 Å. During analysis the Al foil cathode substrate was used as an internal standard to correct for sample displacement. This instrument was also used to measure the as-synthesized powder samples in 1.2 mm inner diameter polyimide capillaries.

### 2.5 Neutron Powder Diffraction

The materials were loaded in 6 mm diameter vanadium sample cans at the home institution in an Ar-filled glovebox with 2 g per sample. Loaded sample cans were shipped to Oak Ridge National Laboratory for measurement at the POWGEN instrument. The instrument was set for the 1.5 Å center wavelength chopper frame and high-resolution guide with the samples at 300 K. The measurements were done for 2 h for each sample, using the total incident neutron count to account for the variation in flux over time. Each sample was measured for a total of 4.1 × 10^12^ proton charge at the spallation target.

### 2.6 X-Ray Absorption Spectroscopy

X-ray absorption near-edge spectroscopy (XANES) was measured using an easyXAFS easyXES150 spectrometer. Samples were pressed into thin pellets with 13 mm diameters using poly­(ethylene oxide) as a binder and diluent. The pellets had a mass loading of 12 mg cm^–2^ for the cathode materials and 7 mg cm^–2^ for the reference samples, to optimize their spectra for this instrument. The reference samples were manganese­(III) oxide and manganese­(IV) oxide (99%, Sigma) as well as manganese foil provided by the instrument manufacturer.

### 2.7 Software

Synchrotron data reduction was done in pyFAI.^33^ Rietveld refinement was done in TOPAS Academic V6 using the scripting interface method.^34^ TOPAS scripts are provided in the electronic Supporting Information. Structures and calculated profiles were exported using pdCIFplotter.^35^ Crystal structures were visualized using VESTA.^36^ XANES normalization was done using Athena.^37^ Data plotting for all figures and parabolic fitting for Figure S4 were done in Igor Pro V8.^38^

## 3 Results and Discussion

### 3.1 Structural Characterization

#### 3.1.1 Ion Ordering and Structural Distortion of NaNM

Both X-ray diffraction (XRD) and neutron powder diffraction (NPD) were measured for the as-synthesized NaNM sample. Except for a minor NiO impurity phase, the XRD pattern (Figure 1) shows major reflections that have usually been assigned to a hexagonal unit cell in the *P*6_3_/*mmc* space group (S.G.)^2^ and a series of weak superstructure reflections at *Q* = 1.9 and 2.0 Å^–1^ attributed to the Na-vacancy ordering.^24,39^ These peaks at *Q* = 1.9 and 2.0 Å^–1^ as well as the faint reflections at 0.9, 1.8, 2.2, 2.9, and 3.1 Å^–1^ were accounted for previously by Lee et al. with a 2√3*a* × 2√3*a P*6_3_ supercell.^39^

**Figure 1:**
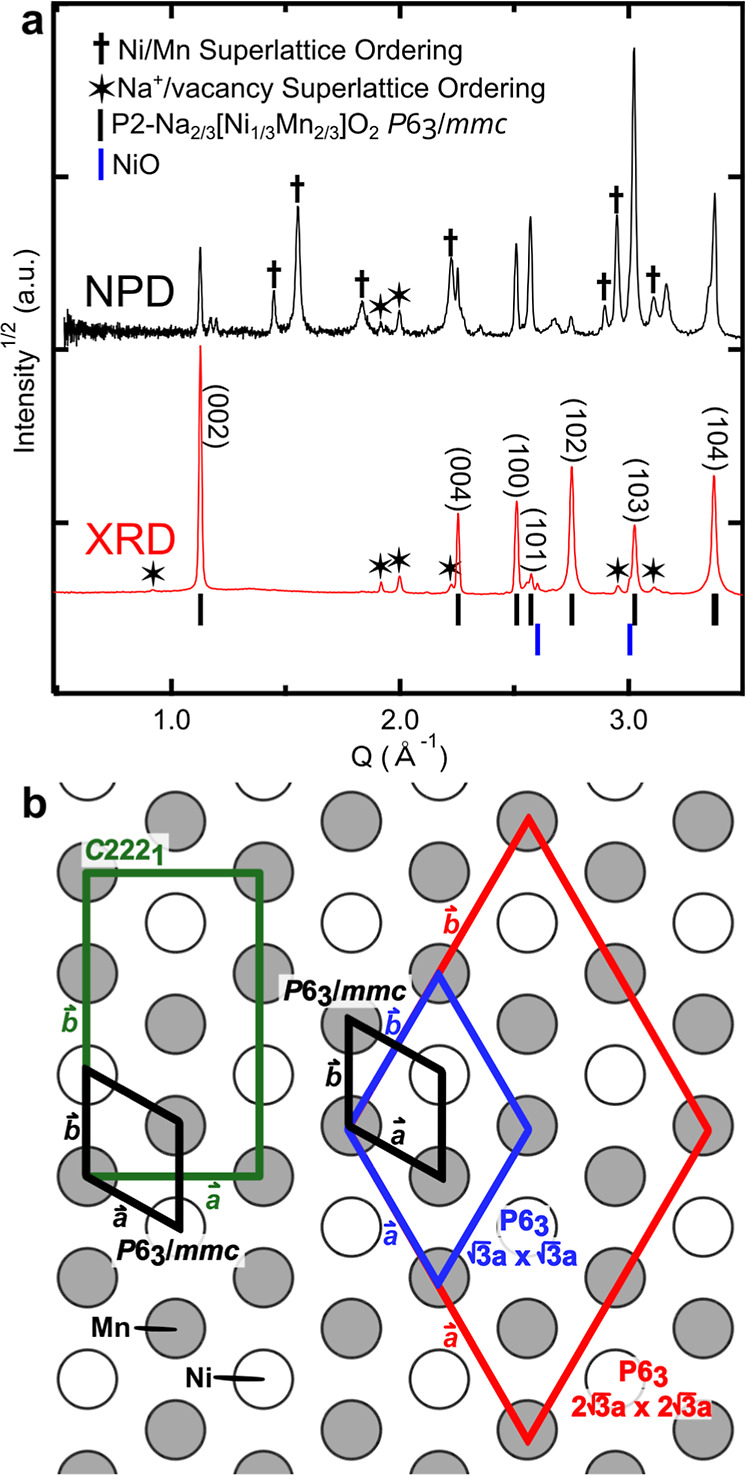
(a) Powder X-ray (red) and neutron (black) diffraction patterns measured for the as-synthesized Na_2/3_[Ni_1/3_Mn_2/3_]­O_2_ sample. Daggers (†) and asterisks (*) indicate the reflections due to the superlattice formed by Ni/Mn and Na^+^/vacancy ordering, respectively. (b) Illustration of the various unit cells considered for NaNM. This view is projected along the [001] direction and overlaid on a transition metal ion layer showing the honeycomb ordering of Ni and Mn ions. The higher symmetry *P*6_3_/*mmc* model is unable to describe the transition metal ordering.

The superstructure reflections in XRD and NPD were reported and modeled previously,^28,39^ so Rietveld refinement using already reported unit cells was attempted. Figure S2 shows a comparison between the highest symmetry S.G. *P*6_3_/*mmc* unit cell known as the standard P2 structure, and the fit using a 10 Å wide *P*6_3_ supercell. The high symmetry cell is a good fit for all of the most intense reflections, however, the weak reflections are all forbidden. The lower symmetry supercell is able to index all of the minor reflections, especially those at *Q* = 0.9, 1.8, 1.9, 2.0, and 2.2 Å^–1^. Unfortunately, it also allows indices for a large number of reflections which have zero intensity in the experimental data. This indicates that the unit cell may not be the lowest possible symmetry which describes the data fully. The *P*6_3_ cell has 10 unique Na sites with independent occupations, so the Rietveld refinement entails a semirandom search for the best mixture of these 10 numbers that can match the Na order peaks in the data. The powder XRD method cannot yield a unique result for this problem with a high degree of certainty. The pool of similar solutions is vast, and there is not enough information given by these few peaks. Additionally, there is the issue of the least-squares minimization routine which prioritizes the most intense features. The low intensity of the relevant Na-vacancy superlattice peaks gives them little influence on the refinement. Single crystal X-ray or electron diffraction would provide a more conclusive analysis for the Na arrangement, which based on powder XRD is not precisely the same as prior reports.

The NPD pattern (Figure 1) shows additional reflections not observed in the XRD pattern. These additional reflections have been attributed to the honeycomb ordering of the Ni and Mn ions in the transition metal layer, which can be described by a hexagonal √3*a* × √3*b* supercell with its origin at the Ni sites (Figure 1b).^40,41^ The symmetry of this unit cell is lower than the *P*6_3_/*mmc* unit cell to allow for TM ordering, which gives rises to the additional peaks observed in the NPD patterns.

Initial attempts to fit both the XRD and NPD patterns show systematic misfits for the (*hk*0) reflections, which is illustrated by the (21̅0) reflection (Figure 2a). Such misfits indicate a distortion from the hexagonal unit cell, which has been observed in a prior NPD, XRD, and electron diffraction study of NaNM.^24^ Yet, the origin of this distortion remains unknown. While such a lattice distortion can be induced by a high concentration of the Jahn–Teller active Mn^3+^ ions, the observed distortion here cannot be explained by Jahn–Teller distortion due to the low concentration of Mn^3+^ in the structure (primarily due to defects and slight off-stoichiometry). For example, the hexagonal unit cell is maintained for the P2–Na_0.59_Mn_0.8_O_2_, where 0.16 Mn per f.u. is in the Jahn–Teller active +3 oxidation state.^42^ Lowering the symmetry to an orthorhombic cell (S.G. *C*222_1_) explains the peak splitting and results in an improved fitting of the experimental peak profile (Figure 2c,d). The orthorhombic cell (*C*222_1_) is related to the hexagonal cell (*P*6_3_/*mmc*) through the following transformation

1 $${\left(\begin{array}{l} a\\ b\\ c \end{array}\right)}_{C222_{1}}=\left(\begin{array}{lll} 2 & 0 & 0\\ 3/2 & 3 & 0\\ 0 & 0 & 1 \end{array}\right){\left(\begin{array}{l} a\\ b\\ c \end{array}\right)}_{P6_{3}/mmc}$$

This distortion manifests as expansion along the [110]_hex_ direction and compression along the perpendicular [1̅10]_hex_ direction or an opening up the hexagonal angle in a monoclinically distorted unit cell. The refined structure shows an equivalent monoclinic angle of 120.154(5)°. This is nearly equivalent to the result from Pfeiffer et al. which reported an equivalent angle of 120.163(5)° for P2–Na_2/3_[Ni_1/3_Mn_2/3_]­O_2_.^22^ In comparison, a typical monoclinic angle for Jahn–Teller-distorted orthorhombic P′2 Mn-rich Na_2/3_[Ni_*z*_Mn_1–*z*_]­O_2_ (*z* = 0.1) is 122.34°,^43^ an order of magnitude larger distortion than observed here.

**Figure 2:**
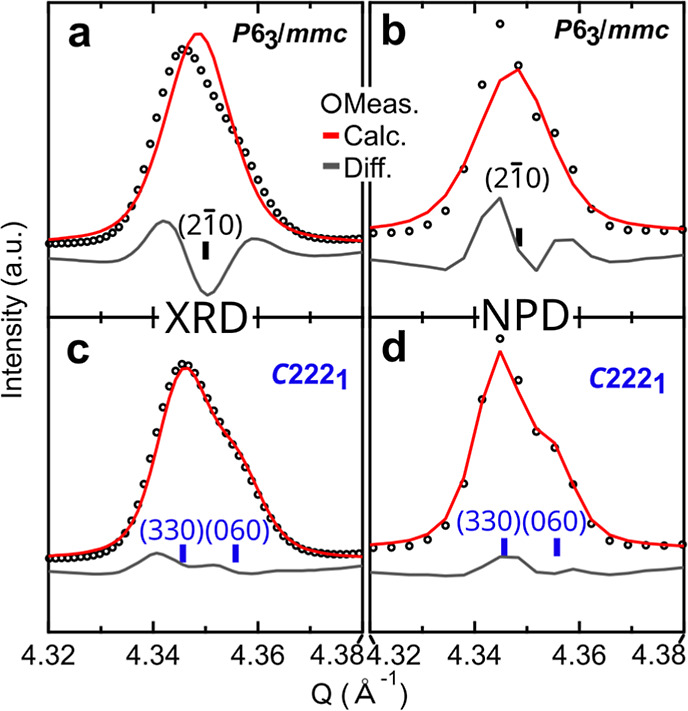
Fitting profile for both the XRD and NPD Bragg peak intensities at *Q* = 4.35 Å^–1^ (a,b) with a single (2̅10) reflection by the hexagonal unit cell (S.G. *P*6_3_/*mmc*) and (c,d) with (330) and (060) reflections by the orthorhombic unit cell (S.G. *C*222_1_).

Based on the analysis of the XRD and NPD patterns measured for the as-synthesized NaNM, both Na-vacancy and Ni–Mn orderings are necessary to describe the real crystal structure. However, Na-vacancy ordering is difficult to account for and does not contribute to significant scattering intensities in either the XRD or NPD pattern. Therefore, we adopt a Na-vacancy disordered structure model in S.G. *C*222_1_ that only considers the Ni–Mn ordering for the combined structure refinement against both the XRD and NPD patterns. However, the Ni–Mn ordered model tends to overfit the XRD pattern, yielding unrealistic site occupancies. To overcome this problem, Ni and Mn ions are disordered on the transition metal sites when modeling the XRD pattern with an additional constraint to keep their average occupancies identical with the Ni–Mn ordered model for the NPD pattern. The XRD and NPD patterns also showed anisotropic broadening of the (13*L*) and (20*L*) reflections with *L* > 0. This broadening was minor so the peak profile was modeled with an empirical anisotropic Lorentzian spherical harmonics component. These modifications yield satisfactory fitting for both the XRD and NPD patterns (Figure S6).

This result was obtained by adding additional constraints in the refinement process: the Ni/Mn ratio of the P2 phase was constrained to be equal to the value obtained by ICP-OES (Table S1)­ since there was no significant impurity phase with Ni or Mn or amorphous phases. The unit cell contains 3 TM sites, and in this case sites 1 and 2 are not distinguished, so their occupation was set to be equal. The “Site 1 + 2” occupation accounts for 2/3 of the TM sites and “Site 3” accounts for 1/3. The “Na total” accounts for the edge-sharing and face-sharing Na sites. The refined structure shows a predominant Mn occupation of 0.97(16) at the Mn rich site, and a predominant Ni occupation of 0.94(3) at the Ni-rich site. This result is similar to a prior study by Pfeiffer et al., which measured the TM site occupation with 0.86 Mn on site 1 and 1 Mn on site 2 for an average of 0.93 at the Mn-rich sites, and 0.92 Ni on site 3.^22^ The complete refined structure parameters are provided in Table S2.

**Figure 3:**
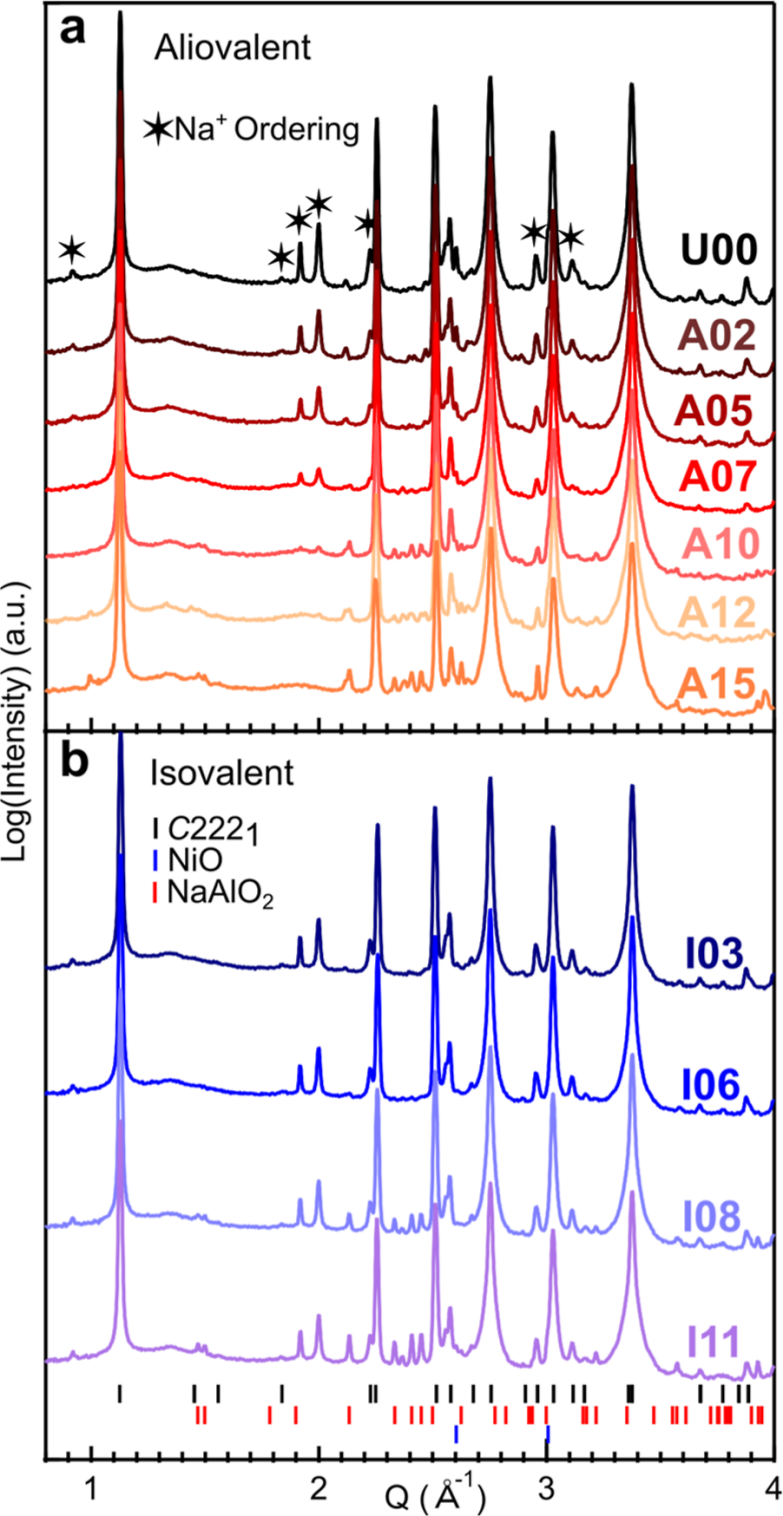
Synchrotron XRD patterns for (a) aliovalent and (b) isovalent-substituted samples. Asterisks (*) on U00 XRD indicate reflections from Na^+^-vacancy ordering.

#### 3.1.2 Effects of Al-Substitution

##### 3.1.2.1 XRD and Na-Vacancy Ordering

The XRD patterns measured for the Al-substituted NaNM are shown in Figure 3. The samples all showed the same major P2 phase. The peaks for the cubic NiO phase impurity decrease in intensity with increasing ICP Al content and disappear at *y* = 0.065 (A07) for the aliovalent-substituted NMA and *y* = 0.028 (I03) for the isovalent-substituted NMA. Meanwhile, a minor orthorhombic NaAlO_2_ phase starts to appear for *y* > 0.065 (A07) for aliovalent and *y* > 0.074 (I08) for isovalent-substituted samples. The formation of an Al-containing impurity phase suggests the difficulty of doping a high concentration of Al in the P2 phase.

The superstructure reflections corresponding to the Na-vacancy ordering decrease in intensity for aliovalent samples as the ICP Al content increases and disappear at A10. This shows the suppression of Na-vacancy ordering with aliovalent Al-substitution. In contrast, the superstructure reflections persist for the isovalent samples regardless of the intended Al doping concentration.

To quantify the extent of Na-vacancy (dis)­ordering, single peak fitting was performed for the prominent Na-vacancy superstructure (12̅0) and (12̅1) reflections at *Q* = 1.92 and 2.00 Å^–1^, respectively. The peak intensity alone can be used to measure the degree of order within the Na layers. Figure 4 shows the intensities of both peaks relative to the (004) reflection. The relative intensity of the (12̅1) reflection decreases nearly linearly with increasing Al concentration for the aliovalent-substituted samples and drops to 0 at *y* = 0.09. A similar behavior is observed for the weaker (12̅0) reflection. This result demonstrates that the aliovalent substitution fully disrupts the Na-vacancy ordering for *y* > 0.09 mol % ICP Al. In contrast, the relative intensity of the (12̅1) reflection for the isovalent-substituted samples is always higher than the aliovalent substituted one for all Al concentrations and decreases from 0.074 to only 0.060 for *y* > 0.02 ICP Al. This result shows that the isovalent substitution does not disrupt the Na-vacancy ordering as much as the aliovalent substitution does or the ICP overestimates the doped Al concentration in the P2 crystal lattice or both. This observed disruption of Na-vacancy ordering with elemental substitution at the transition metal sites is consistent with previous elemental substitutional studies on materials based on Na_2/3_[Ni_1/3_Mn_2/3_]­O_2_, where the Na^+^-vacancy ordering reflections disappear in the substituted samples.^24,28,44^ For example, Kubota et al. demonstrated that the Na^+^-vacancy ordering XRD peaks are suppressed in Na_2/3_[Ni_1/3_M*n*_1/2_Ti_1/6_]­O_2_ and fully eliminated in Na_2/3_[Ni_1/3_Mn_1/3_Ti_1/3_]­O_2_.^45^

**Figure 4:**
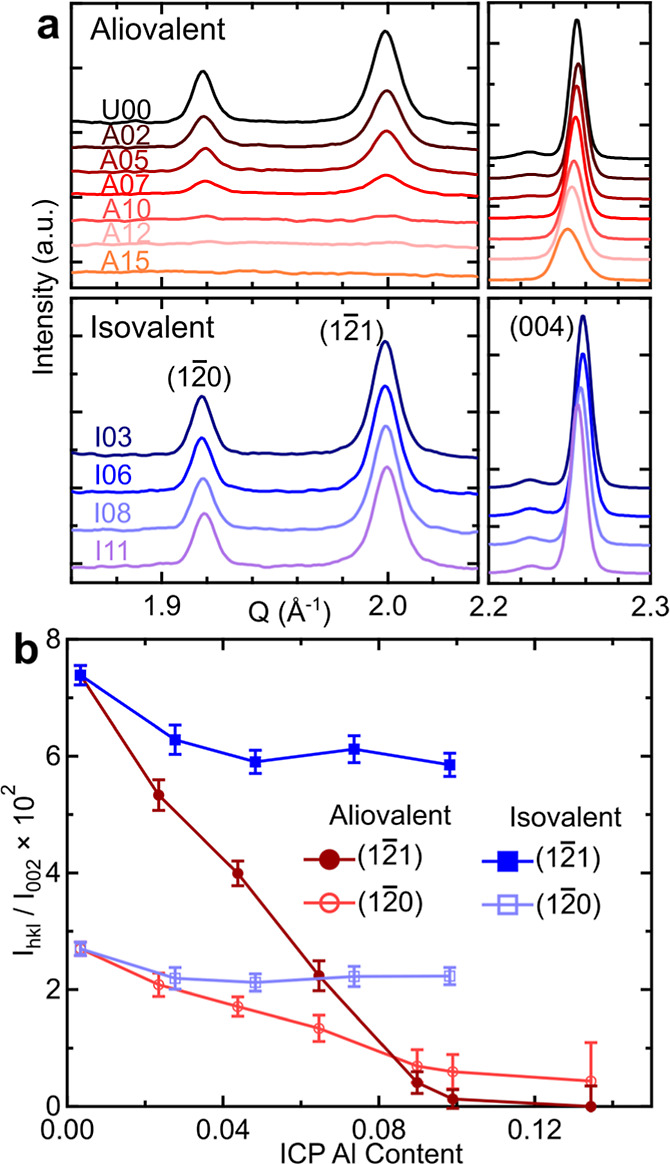
(a) Select Na^+^-vacancy ordering XRD reflections indexed to (12̅0) and (12̅1) in the orthorhombic unit cell (S.G. *C*222_1_). These are compared to the (004) reflection to show the relative intensity changes. (b) Integrated intensity of the Na^+^-vacancy ordering superlattice reflections in XRD normalized by (002).

##### 3.1.2.2 NPD and Transition Metal Site Occupation

The NPD patterns for NaNMA (Figure 5) show significant changes in the superstructure reflections from the honeycomb Ni–Mn ordering. The aliovalent samples have a drastic decrease in the intensities of the reflections produced by the Ni–Mn ordering (e.g., reflections near 1.4 and 1.6 Å^–1^ in Figure 5) with increasing Al concentration. This is evidence of changes in the transition metal occupancy because of Al-substitution in the crystal lattice. In contrast, the isovalent samples show little change in their superlattice reflection intensities.

**Figure 5:**
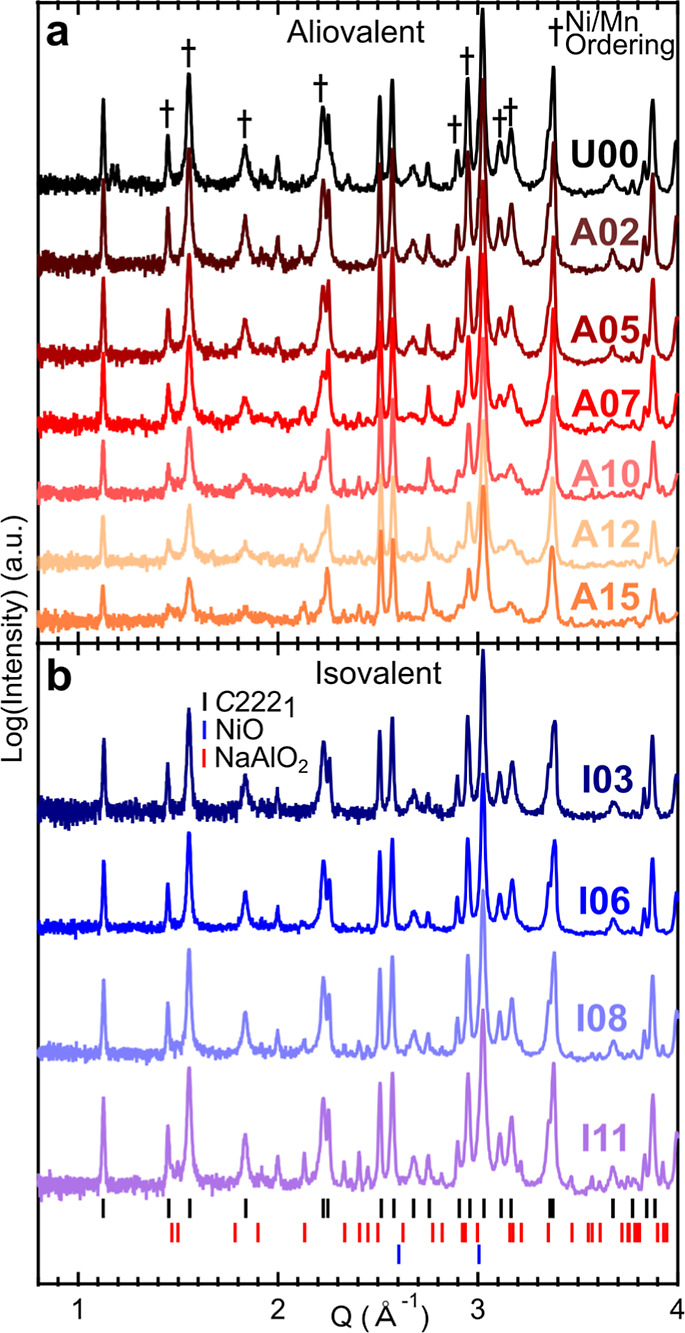
NPD patterns for (a) aliovalent and (b) isovalent-substituted NaNMA. Daggers (†) indicate reflections from Ni/Mn honeycomb ordering.

##### 3.1.2.3 Al-Substituted Structure via Combined XRD and NPD Analysis

To quantify the structural changes upon Al-substitution, Rietveld refinement was conducted simultaneously against both the XRD and NPD patterns for all substituted samples. The refined lattice parameters for all samples are shown in Figure 6 and all refined structure parameters are listed in Tables S2–S12. For the orthorhombically distorted samples, the half diagonal of the *ab* face (denoted as *b*′) is equivalent to the *b* lattice parameter of the undistorted hexagonal unit cell, and the angle between the *a* axis and the *ab* face diagonal (denoted as γ′) is equivalent to the γ angle of the undistorted hexagonal unit cell. Therefore, the refined lattice parameters of the distorted orthorhombic cell are transformed to a pseudohexagonal cell via eq 2 to show the extent of the orthorhombic distortion, which is characterized by the discrepancy between *a*′ and *b*′ and the deviation of γ′ from 120°.

2 $${\left(\begin{array}{l} a^{\prime }\\ b^{\prime }\\ c^{\prime } \end{array}\right)}_{Pseudohex.}=\left(\begin{array}{lll} 1 & 0 & 0\\ -1/2 & 1/2 & 0\\ 0 & 0 & 1 \end{array}\right){\left(\begin{array}{l} a\\ b\\ c \end{array}\right)}_{Orthorhombic}$$

For the aliovalent substituted samples, the difference between *b*′ and *a*′ decreases while γ′ decreases and approaches 120° as the ICP Al content increases to 0.065 per f.u. of NaNMA. For ICP Al content above 0.065, the orthorhombic distortion is not observed. The average of the *a*′ and *b*′ lattice parameters of the distorted phase and the *a* lattice parameters of the undistorted phase decrease monotonically as the ICP Al content increases to 0.099 per f.u. of NaNMA. Further increase of the Al content does not lead to a substantial change of the *a* lattice parameter. The *c* lattice parameter initially decreases from 11.146(2) Å to 11.143(4) Å with 0.02 ICP Al and increases monotonically to 11.171(7) Å as the ICP Al content increases to 0.13. This trend of decreasing *a* lattice parameter and increasing *c* lattice parameter is consistent with previous studies, as Hasa found *a* decreased to 2.8737 Å and *c* increased to 11.203 Å in Na_0.6_[Ni_0.22_Al_0.11_Mn_0.66_]­O_2_.^17^

**Figure 6:**
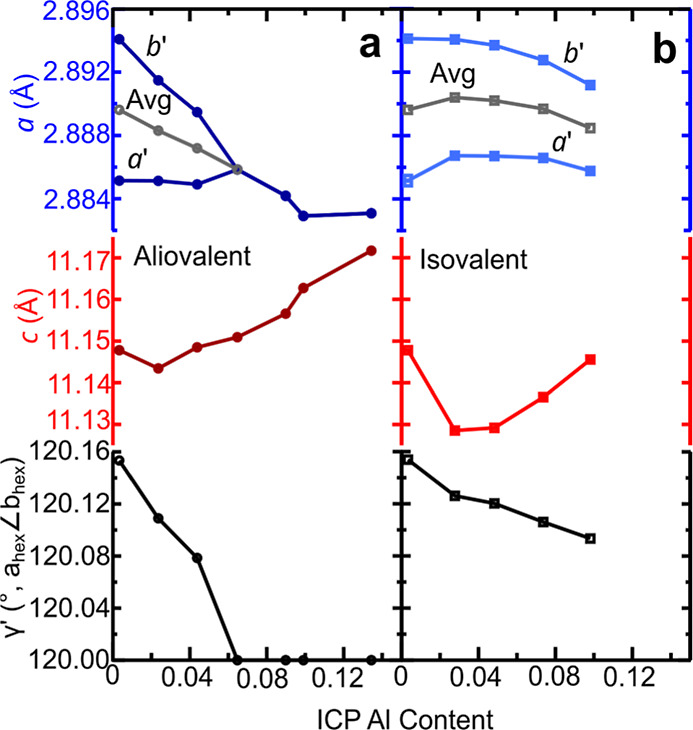
Refined lattice parameters against synchrotron XRD patterns measured for (a) aliovalent and (b) isovalent-substituted NaNMA samples. The *a*′, *b*′, and γ′ lattice parameters are defined for a pseudo hexagonal lattice according to eq 2 for samples with orthorhombic distortion. The estimated standard deviations of the refined values are within ±0.001% of the refined values.

For the isovalent substituted samples, *b*′ decreases monotonically with increasing Al content while *a*′ increases from 2.8851(5) Å to 2.8867(3) Å upon initial 0.028 ICP Al-substitution, remains effectively constant between 0.028 and 0.074 ICP Al before decreasing to 2.8857(6) Å at 0.098 ICP Al content. As the ICP Al content increases from 0 to 0.098, the difference between *a*′ and *b*′ decreases only from 0.0089(7) to 0.0054(3) Å and the γ′ angle decreases only slightly from 120.154(5) to 120.093(6)°. Meanwhile, the *c* lattice parameter initially drops to 11.129 Å at 0.028 ICP Al content and increases to 11.145(6) Å until the ICP Al content of 0.098. The disparate and inconsistent changes in the *a*′, *b*′ and *c* lattice parameters render them poor descriptors for the extent of Al-substitution in the P2 phase.

An interesting observation is that the disappearance of the lattice distortion coincides with the elimination of the Na^+^-vacancy ordering at ICP Al content of 0.065, suggesting a correlation between the two structural features. Despite the lack of an experimental solution for the Na^+^-ordering scheme in the P2–Na_2/3_Ni_1/3_Mn_2/3_O_2_ phase, first-principles simulations suggest a plausible zigzag ordering of Na^+^ ions^39^ that partially explains the observed Na^+^-vacancy ordering superstructure reflections.^29,45^ The zigzag ordering pattern may also be described in a rectangular lattice which is compatible with the observed orthorhombic distortion. Therefore, Na^+^-vacancy ordering likely drives the minor lattice distortion.

To refine the mixed transition metal site occupancies, the combined XRD and NPD Rietveld refinement was conducted with additional constraints: Since there were no impurity phases containing Ni or Mn, the Ni/Mn ratio in the layered phase was constrained to the Ni/Mn ratio obtained by the ICP analysis. The refined site occupancies for the substituted samples are summarized in Figure 7 and charge-balanced chemical formulas based on these values are provided in Table S13. For the aliovalent-substituted samples, the Na occupancy remains effectively constant at 0.681(7) until ICP Al content increases to 0.065, and then decreases to 0.618(6) Na when the ICP Al content further increases to 0.134. The refined Al composition matches the target one as the ICP Al content increases to 0.065. With further increase of the ICP Al content, the refined Al composition still increases but trails the ICP value, which shows a leveling effect for incorporating a higher concentration of Al in the P2 phase. This divergence from the ICP Al content coincides with the appearance of the NaAlO_2_ impurity (Figure S18), which accounts for the remaining Al not included in the P2 phase. Regardless of the ICP Al content, Sites 1 and 2 are almost exclusively occupied by Mn. In contrast, Site 3 becomes increasingly occupied by both Al and Mn at the expense of Ni as the ICP Al content increases. This refinement result demonstrates the success of the aliovalent substitution with Al in the layered phase. This also helps explain the extinguished Na^+^-vacancy ordering reflections: the charge of the TM superlattice is more uniform with Al^3+^ in these sites than Ni^2+^, so the driving force for Na^+^ ions to order is decreased.

**Figure 7:**
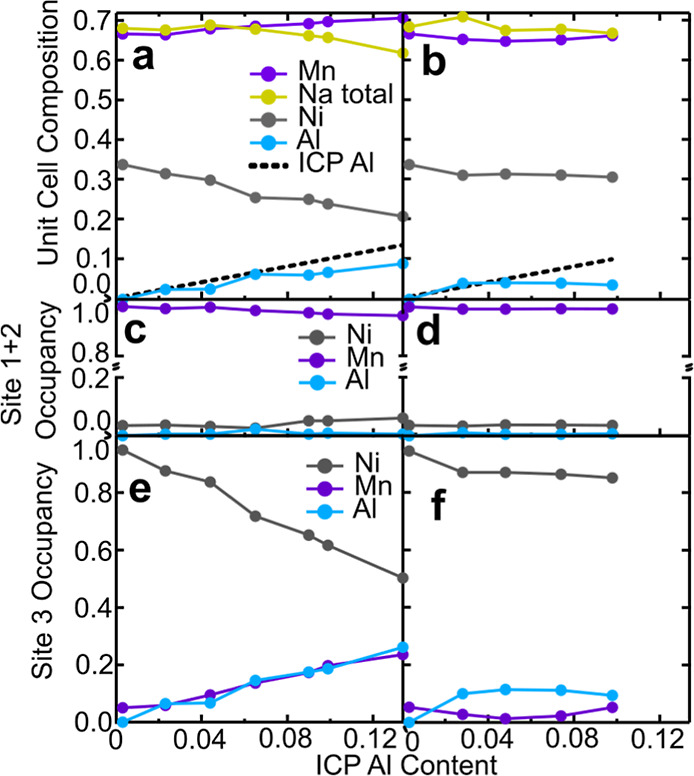
(a,b) Refined metal stoichiometry and (c–f) Ni, Mn, and Al site occupancies for (a,c,e) aliovalent and (b,d,f) isovalent-substituted NaNMA samples. The dashed lines in a and b correspond to the measured ICP Al content as a reference.

For the isovalent-substituted samples, the refined Al composition is nearly the same as the ICP Al content for the sample with the ICP Al content of 0.028 and becomes nearly constant in samples with higher ICP Al content. Like the aliovalent-substituted samples, Al was found preferentially at the Ni-rich site (Site 3), rather than equally distributed on all the transition metal sites. Therefore, the isovalent substitution of both Ni and Mn with Al is not successful.

The refinement results demonstrate a strong driving force for Al^3+^ to substitute Ni^2+^ regardless of the precursor stoichiometry. This outcome can be explained by considering the size disparities between Ni^2+^ Mn^4+^, and Al^3+^ with crystal radii of 0.83, 0.67, and 0.68 Å respectively:^46^ the larger Ni^2+^ ion is under compressive strain in the lattice, which can be relieved by the substitution with the smaller Al^3+^ ion. This size effect prevails over the electrostatic effect that is expected to favor Al^3+^ substitution of Mn^4+^ by reducing electrostatic repulsion between the nearest transition metal ion neighbors. The size effect also explains why the isovalent substitution is more difficult than aliovalent substitution: substitution of Ni^2+^ by either Al^3+^ or Mn^4+^ relieves the lattice strain while substitution of Mn^4+^ by Al^3+^ does not.

##### 3.1.2.4 Oxidation of Mn in Al-Substituted Structures

To examine the charge balance of the Al-substituted materials, the oxidation state of Mn was measured via XANES at the Mn K-edge. The synthesis procedure used manganese­(III) oxide as a manganese precursor, which was intended to be oxidized to Mn^4+^ at 1000 °C under air, yielding NaNMA containing Mn^4+^. Several previous studies have reported that NaNM has this formal Mn^4+^ in the as-synthesized state from XANES as the Mn K-edge of NaNM appears at the same energy as manganese­(IV) oxide.^5,47,48^ XANES spectra of NaNMA are shown in Figure 8a. The edge energy was identified through the first derivative of the absorption coefficient (Figure 8b) with the absorption edge energy at the maximum value of this derivative. This analytical method has been used previously in studies on substituted P2 NaNM^49,50^ and Li­[Ni_*x*_Mn_*y*_Co_*z*_]­O_2_ ^51,52^ cathode materials. The Mn K-edge energy noted as E_1_ is aligned with the edge energy for manganese­(IV) oxide at 6558.0 eV. Quantitative determination of the edge energies of NaNMA was done by fitting the peaks in the first derivative, shown in Figure 8c. All of the NaNMA samples had Mn K-edge energies within 0.2 eV of the manganese­(IV) oxide reference with random variation between samples due to the spectrometer resolution and noise. This confirms that the oxidation state of Mn in the NaNMA samples is Mn^4+^ regardless of the aluminum content.

**Figure 8:**
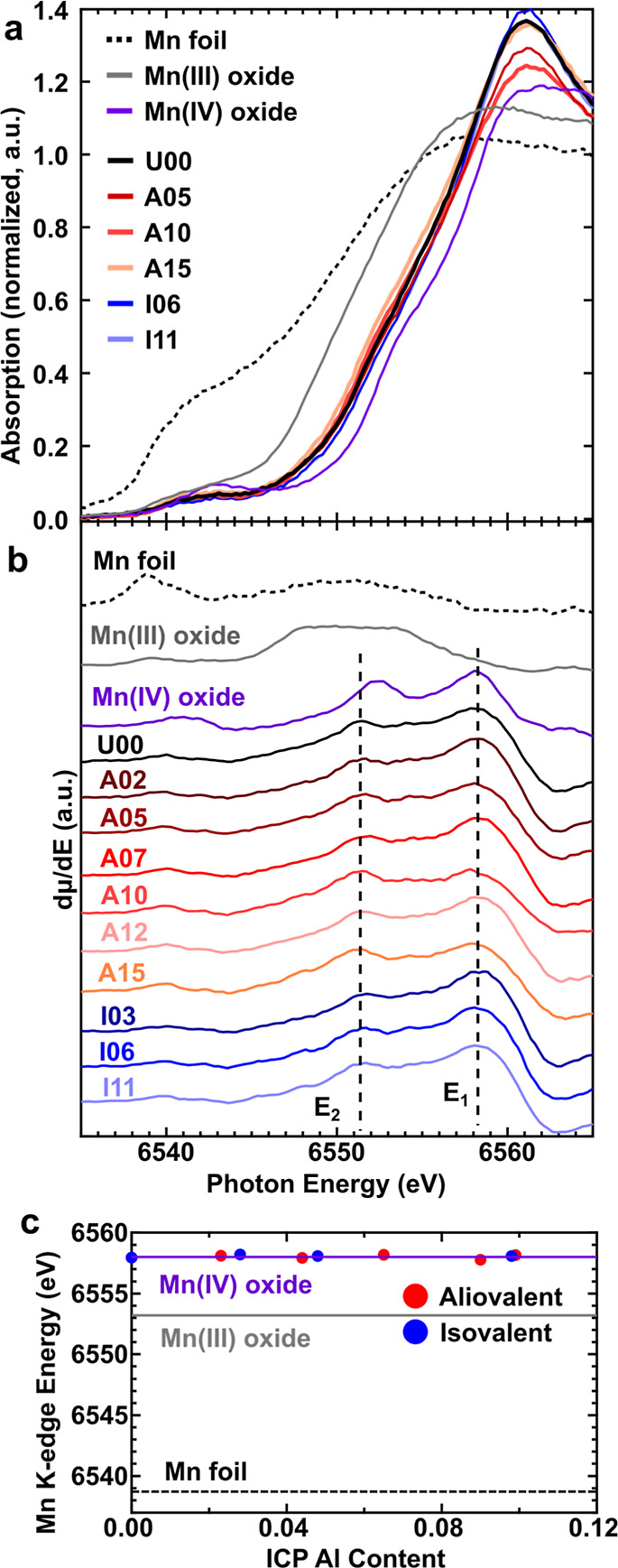
(a) XANES at the Mn K-edge for reference materials and NaNMA. (b) First derivative of absorbance with respect to energy. Maxima indicate inflection points in μ­(E). The energy of the inflection points in the NaNMA samples are indicated by lines E_1_ and E_2_. Energy E_1_ is aligned with the edge energy of manganese­(IV) oxide, indicating NaNMA contains Mn^4+^. (c) Edge energies for NaNMA were measured by fitting the E_1_ region of dμ/dE with a Gaussian peak. These are compared with the edge energies of the reference materials.

Aliovalent-substituted NaNMA has formal formulas following Na_2/3_^+^[Ni_1/3*y*_^2+^Mn_2/3_^4+^Al_*y*_^3+^]­O_2_^2–^ which results in one excess positive charge per Al ion. For these materials to follow charge-neutrality this excess positive charge must be compensated by equal negative charge. One option for this negative charge is Mn^4+^/Mn^3+^ reduction (Figure 8). A linear regression between the Mn K-edge energy of manganese­(III) oxide and manganese­(IV) oxide results in a slope of 0.05 eV per reduced Mn ion. With a hypothesized charge-balanced formula of ${Na}_{2/3}^{+}\left[{Ni}_{1/3-y}^{2+}{Mn}_{2/3-y}^{4+}{Mn}_{y}^{3+}{Al}_{y}^{3+}\right]O_{2}^{2-}$ the refined Al content of *y* = 0.058 in A07 would produce a Mn K-edge energy shift of 0.4 eV. This shift was not observed in our XANES data, with no downward energy trend in the Mn K-edge energies, however this expected shift is small and may not be easily reproduced with laboratory XANES. Another possibility for charge balance is Ni^2+^/Ni^1+^ reduction, which is at a lower potential than Mn^4+^/Mn^3+^ reduction so is not energetically favored. With regards to charge compensation by the formation of point defects, there may be sodium deficiency. Na off stoichiometry is not observed for aliovalent samples with ICP Al < 0.08, but is observed for aliovalent samples with ICP Al > 0.08 (Figure 3a). An additional point defect option is transition metal vacancy, which is unstable in this structure. It is currently not known what the charge compensation mechanism is for the ICP Al < 0.08 as-synthesized aliovalent-substituted NaNMA, but partial Mn reduction is the most plausible explanation.

Isovalent-substituted NaNMA exhibited limited Al occupation at the TM site. The refined compositions of these materials (Figure 7b) with Al occupying only the Mn site and not the Ni site, suggests these materials are not simply “isovalent” as intended with a nominal formula of Na_2/3_^+^[Ni_1/3‑*y*/2_^2+^Mn_2/3‑*y*/2_^4+^Al_*y*_^3+^]­O_2_^2–^. Therefore, charge compensation is required for charge neutrality as a result of Al-substitution. The isovalent samples do not show significant sodium deficiency, nor do they have a measurable Mn reduction (Figure 8). It is likely that the 3–4 mol % Al-substituted is charge compensated by a partial Mn reduction, which is too small to resolve with XANES (the induced Mn K-edge energy shift by 3–4 mol % Al-substitution would only be 0.3 eV).

##### 3.1.2.5 Impact of Al-Substitution on Stacking Faults

Anisotropic broadening of the (13*L*) and (20*L*) reflections with *L*> 0 is observed for all samples (Figure 9) and is attributed to stacking faults between the TMO slabs. The samples trended toward greater broadening in their (13*L*) and (20*L*) reflections with increasing ICP Al content, which indicates increased concentration of stacking disorder, such as the intergrowth of P2 and P3 ^22,53^ or O2 layers,^9^ with higher Al content. Since the interslab spacing is significantly contracted for O2 structures, this type of stacking fault would result in the broadening of the (00*l*) reflections, which is not observed here. In comparison, the P3 structure often competes with the P2 structure at high temperatures with Na contents of 2/3 per f.u.,^23,53–55^ while the O2 phase is found in desodiated (charged) compositions.^9^ Therefore, stacking faults with only the P3 layer insertions is considered in the present case.

**Figure 9:**
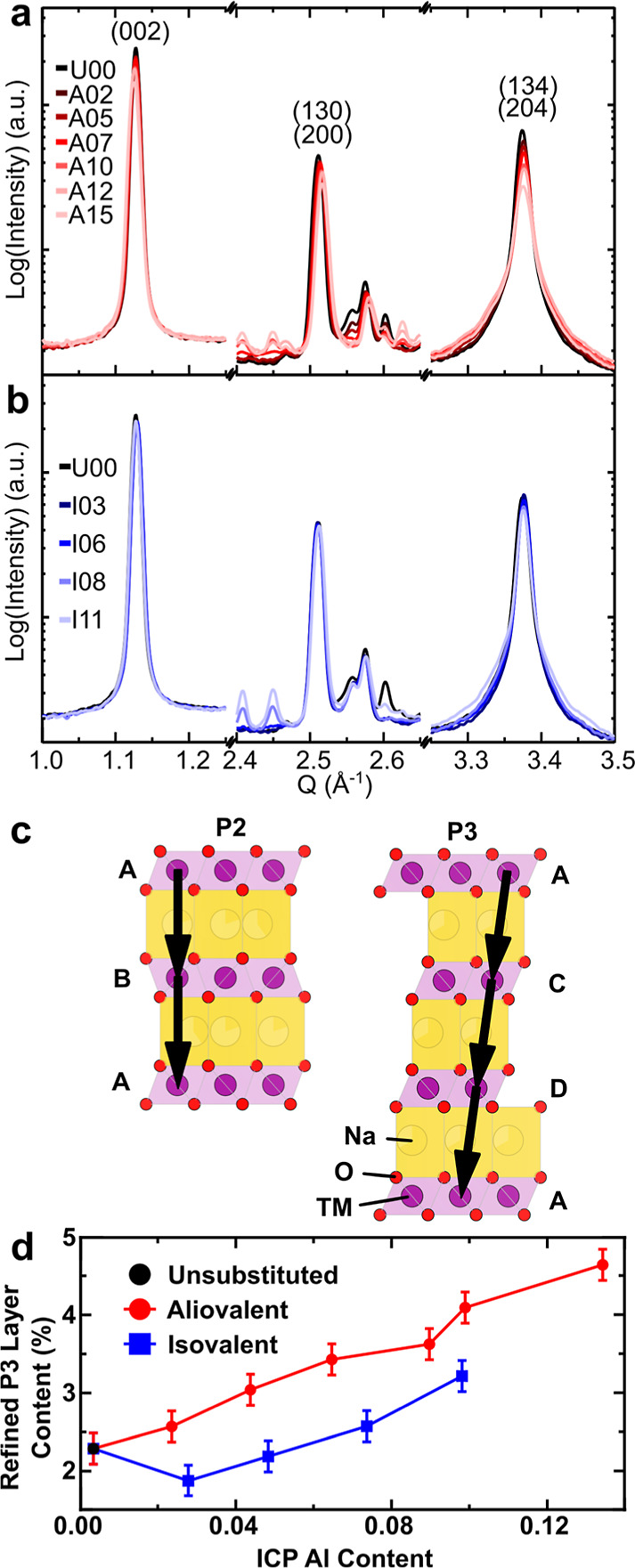
Intensity profiles of select (13*L*) and (20*L*) with *L* > 0 vs. (*hk*0) and (002) reflections for (a) aliovalent and (b) isovalent-substituted samples. The intensity is plotted in logarithmic scale to emphasize the Lorentzian-like broadening profile. Profiles for the unsubstituted sample (U00) are shown as reference. (c) Schematic illustration of P2 and P3 stacking of TMO layers. (d) P3 layer stacking fault concentrations determined by Rietveld refinement against the XRD patterns for both Al-substituted and nonsubstituted samples.

To quantify the stacking fault concentration, XRD patterns were simulated for structures with various stacking fault probabilities and compared with the measured patterns. The stacking faults were modeled using a supercell approach in TOPAS Academic with 200 layers of P2 and varying concentrations of P3 stacking inserted. During this analysis the site occupancies and *a* and *c* lattice parameters were refined. The fault probability corresponds to the stacking fault concentration with the lowest R_wp_, which is determined through a curve fitting approach. The stacking fault probability that yields the best fit to the measured pattern was considered to be the measured concentration of P3 layer intergrowth. Examples of these calculated patterns and analyses are shown in Figures S3 and S4. The measured stacking fault concentrations are shown in Figure 9d. The unsubstituted sample shows 2.3 ± 0.2% P3 layers. The aliovalent-substituted samples show a linear correlation between ICP Al content and stacking fault concentration, increasing to 4.6 ± 0.2% for A15 (0.13 ICP Al content). The isovalent samples also show a linear relationship between stacking fault concentration and the ICP Al content with a similar slope to the aliovalent-substituted samples. The stacking fault concentration is found to be lowest at 1.9 ± 0.2% for Sample I03 and increases to 3.2 ± 0.2% for I11. The stacking fault concentration for the isovalent-substituted samples is systematically lower than the aliovalent-substituted samples by 1%.

One explanation for this increase in stacking disorder with Al content could be that Al increases the temperature of the P2/P3 phase boundary. The P3 phase of Na_2/3_[Ni_1/3_Mn_2/3_]­O_2_ is favored at lower temperatures, typically 600 to 700 °C^54,55^ versus 900 °C or higher for a typical P2 phase.^4,22,39,40,56^ In our preliminary synthesis attempts at 900 °C the Al-substituted materials contained a mixture of P2 and P3 phases (Figure S17), which was the rationale for raising the temperature to 1000 °C. This trend of increasing temperature for the P2/P3 phase boundary with increasing Al content in NaNMA was also observed by Kumar et al.^57^

### 3.2 Effect of Al-Substitution on Electrochemistry

Galvanostatic cycling was conducted to characterize the electrochemical behavior of NaNMA. Figure 10 shows the voltage profile for the second charge/discharge cycle, which is informative of the underlying reaction mechanism. See Figure S24 for the first charge/discharge cycle, which may be influenced by irreversible reactions on the interfaces. The nonsubstituted sample shows a high voltage plateau at 4.2 V, corresponding to the O2–P2 phase transition,^9^ and a staircase-like profile between 2.8 and 4 V, which is attributed to the transitions between different Na-vacancy ordering schemes.^58^ For aliovalent substituted samples (Figure 10a), the staircase profile between 2.8 and 4 V becomes increasingly smeared with increasing Al-substitution, indicating the suppression of the Na-vacancy ordering commonly observed in substituted NaNM materials.^28,59^ While the disruption of Na-vacancy ordering smears the staircase voltage profile for Na ion (de)­intercalation in the P2 phase, the change in the first-order P2–O2 phase transition in the high voltage regime is unlikely to be related to this disrupted ordering. The recent work by Komaba et al. on a Ti and Zn dual substituted NaNM showed a drastic change in the high-voltage phase transition to a solid solution mechanism despite the preservation of Na-vacancy ordering.^45^ The voltage profile above 4 V deviates from a flat plateau and becomes increasingly sloped with Al-substitution, which suggests a departure from the classical two-phase reaction between the P2 and O2 phases. In contrast, isovalent substitution does not fully suppress the staircase profile in the low voltage regime and still exhibits a flat profile in the high voltage regime, similar to the nonsubstituted sample. The effect of Al-substitution on the electrochemistry is further elucidated in the differential capacity (dQ/dV) plot, where aliovalent Al-substitution results in more significant broadening of the dQ/dV peaks than isovalent Al-substitution. The lesser effect on the electrochemistry observed for the isovalent-substituted samples is consistent with the limited Al content in the P2 phase regardless of the ICP Al content.

**Figure 10:**
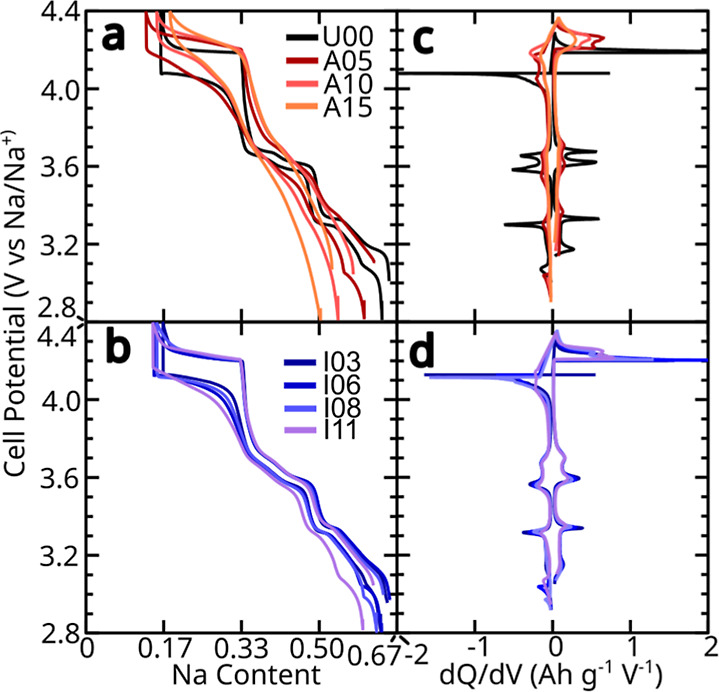
(a,b) Voltage profiles and (c,d) the corresponding differential capacity plot for (a,c) aliovalent and (b,d) isovalent-substituted NaNMA electrode samples during the second charge–discharge cycle at 0.05 C (8.65 mA g^–1^).

Prior work has shown that the electrochemical capacity in NaNM is derived from Mn redox below 2 V, and Ni redox above 2 V.^5,47^ Therefore, it is anticipated that the theoretical capacity corresponding to Ni redox (i.e., assuming transfer of two electrons per Ni ions for the nominal Ni^4+^/N^2+^ redox) would decrease with decreasing Ni content. Since all samples show a substantial irreversible capacity (12–38% for aliovalent-substituted samples and 11–15% for isovalent-substituted ones) during the first cycle, specific capacity in the second cycle is used to compare with the theoretical prediction (Figure 11). The capacity of U00 at. 0.334 ICP Ni content (131 mA h g^–1^) was significantly lower than the theoretical value (173 mA h g^–1^). The voltage profile (Figure 10) indicates that U00 is unable to access the Na composition range of 0 < *x* < 1/6 in Na_*x*_[Ni_1/3_Mn_2/3_]­O_2_. This lower than theoretical capacity was observed for all the samples with over 0.28 ICP Ni content. For aliovalent-substituted samples with more than 0.28 ICP Ni, the reversible capacity remained ca. 130 mA h g^–1^, corresponding to the cycling of Na in the range of 1/6 < *x* < 2/3, despite differences in the Ni content. For aliovalent-substituted samples with lower ICP Ni than 0.28, the capacity decreased linearly, following the theoretical trend. The isovalent-substituted samples showed a linear decrease in capacity with decreasing Ni content, in agreement with the theoretical trend.

**Figure 11:**
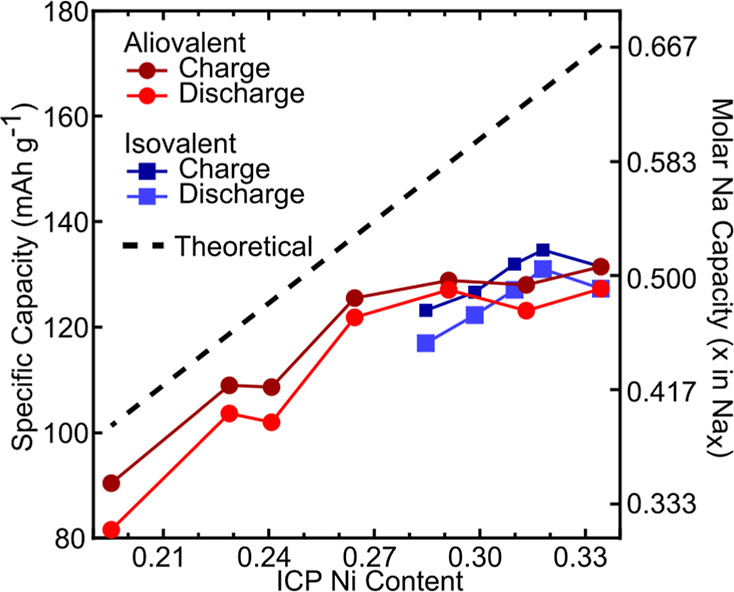
Comparison between the theoretical (assuming Ni^2+/^Ni^4+^ redox) and measured capacity of aliovalent and isovalent-substituted NMA electrode samples. The measured data is obtained from the second charge/discharge cycle to reduce the influence from side reactions during first charge.

Prior publications have claimed that oxygen may be oxidized when NaNM is charged beyond 1/3 Na content per f.u.^13,47,48,54^ A previous X-ray absorption spectroscopic study has identified in the desodiated O2 phase for the stoichiometric Na_2/3_Ni_1/3_Mn_2/3_O_2_ sample a change in the O K-edge spectra, which is attributed to “anionic oxygen redox”.^13^ This O K-edge feature is likely caused by the increased covalency of the Ni–O bond in the desodiated phase.^60^ Since Al preferentially substitutes the Ni site without disrupting the transition metal honeycomb ordering, the local Ni–O bonding environment is preserved, but its concentration decreases at the expense of the Al–O bond with Al-substitution. Therefore, Al-substitution is expected to yield a diminished “anionic oxygen redox” because of the decreased concentrations of Ni–O bonds but not to affect the local Ni–O redox.

Long-term galvanostatic charge/discharge cycling was done with an upper cutoff potential of 4.3 V vs Na/Na^+^, rather than 4.4 V which accesses a greater capacity, to avoid side reactions with the electrolyte. The U00 sample (Figure 12a) had a rapid decrease in its capacity during the first 2 cycles, then continued to lose capacity at a lower rate, retaining 74 mA h g^–1^ at the 50th cycle (52% capacity retention). The capacity retention at the 50th cycle increases with increasing ICP Al content. The retention was gradually improved as the ICP Al content increased in the aliovalent materials, with the A10 sample retaining the highest capacity of 88 mA h g^–1^ at the 50th cycle. Except A12 and A15, all aliovalent-substituted samples outperform U00 in energy density (Figure 12c). The low energy density observed for A12 and A15 is attributed to the lower specific capacity (Figure 12a) in addition to the increased voltage polarization, which is captured by the lower energy efficiency than the other samples (Figure 12e). All isovalent-substituted samples show similar capacity (Figure 12b), energy density (Figure 12d), and energy efficiency (Figure 12f) during the first 50 cycles, comparable to the best results found in the aliovalent-substituted samples. The lack of differentiation between the isovalent-substituted samples is consistent with the structure result that all isovalent-substituted samples show the same level of Al-substitution.

**Figure 12:**
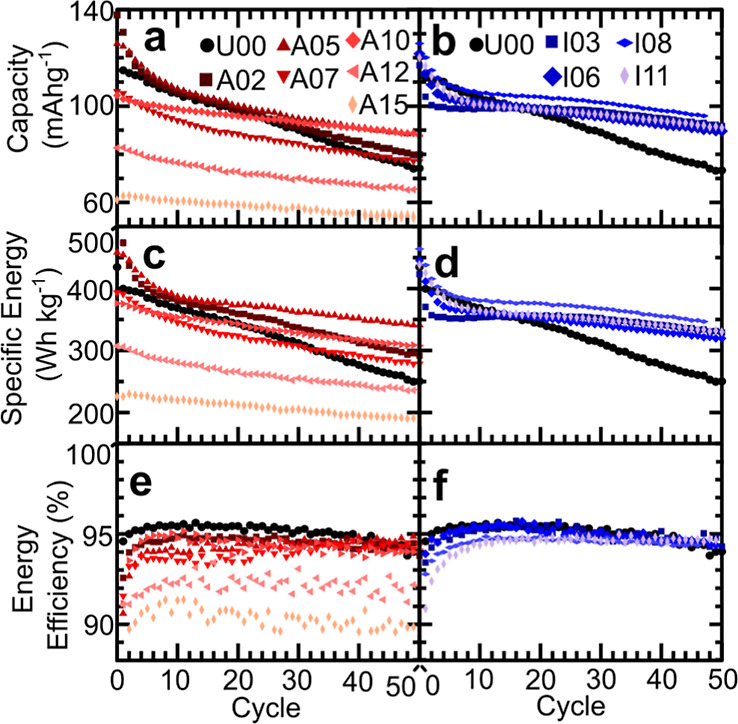
(a,b) Specific discharge capacity, (c,d) specific energy, and (e,f) energy efficiency measured for (a,c,e) aliovalent and (b,d,f) isovalent-substituted NMA electrode samples during galvanostatic cycling between 4.3 and 2.8 V (vs Na metal anode) at 0.1 C (17.3 mA/g).

### 3.3 Effect of Al-Substitution on Structural Phase Transition

Operando X-ray diffraction was done to determine the charge/discharge reaction mechanism in NaNMA and how it deviates from the unsubstituted NaNM. During operando cycling the cells were given a lower cutoff potential of 3.5 V, rather than the typical cutoff voltage of 2.8 V, to examine the high-voltage reaction for multiple cycles during the limited beam time. The >3.5 V region in NaNM has been documented to have poor stability, while the 2.8 to 3.5 V region has a relatively high stability.^19,39^ Rietveld refinement was conducted to obtain the unit cell parameters. For the nonsubstituted U00 sample (Figure 13a), the *c* lattice parameter of the P2 phase increases from 11.140(3) Å at the beginning of charge to 11.359(3) Å at 4.2 V and remains constant during the P2–O2 two-phase reaction, where the *c* lattice parameter collapses to 8.876(3) Å for the O2 phase. Meanwhile, the *a* lattice parameter of the P2 phase decreases to 2.843(2) Å at the end of charge. This observation is consistent with prior studies,^2,9,18^ where a single-phase reaction is observed for the P2 phase upon charging from 3.0 to 4.2 V, followed by the P2–O2 phase transition in the high voltage plateau. Prior research concluded that the P2 phase is stable in the range of 1/3 < *x* < 2/3 (for x in Na_*x*_[Ni_1/3_Mn_2/3_]­O_2_), desodiation below *x* = 1/3 induces the spontaneous transition to an O2 phase,^3^ and sodiation above *x* = 2/3 induces a transition to an orthorhombic P′2 phase.^22^ The electrochemical capacity for this phase change is nearly equal to the theoretical capacity for cycling 1/3 Na per f.u. of Na_*x*_[Ni_1/3_Mn_2/3_]­O_2_, suggesting negligible Na content in the O2 phase.^3^ During the first cycle of the U00 sample (Figure 13a), only 73.7(1.7) wt % of the P2 phase was transformed to the O2 phase at the end of charge. Assuming this P2 phase contains 1/3 Na per f.u. and the O2 phase has negligible Na content, this equates to a total capacity in the high-voltage plateau of 0.247(5) electron transfer per f.u., which is in good agreement with the capacity measured via electrochemistry at 0.233 electron transfer per f.u. The second charging produced 60.3(1.8) wt % of the O2 phase, corresponding to 0.200(6) electron transfer per f.u. from XRD while the electrochemical capacity was only 0.147 electron transfer per f.u.

**Figure 13:**
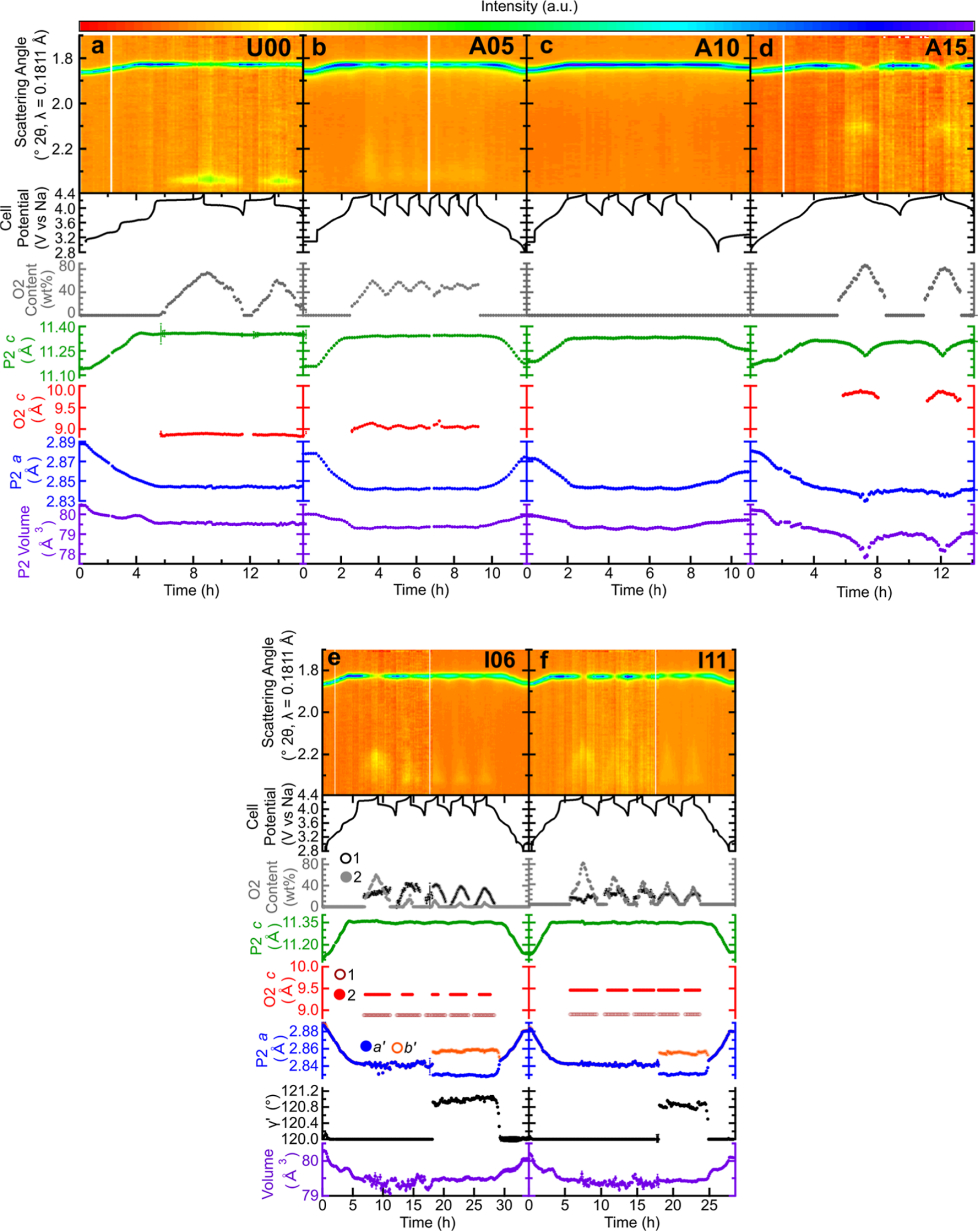
Operando XRD patterns showing the (002) reflection, corresponding voltage profile, and refined unit cell parameters of the P2 and O2 phases during galvanostatic cycling for (a) unsubstituted (U00), aliovalent-substituted (b) A05, (c) A10, and (d) A15, and (e) I06 and (f) I11 electrode samples. For isovalent samples the signal-to-noise ratio was too poor to measure the orthorhombic distortion from 1.3 to 18 h so γ′ was constrained to 120° for this segment. White bands in the XRD patterns correspond to loss of X-ray beam during the beamtime.

For aliovalent-substituted samples (A05, A10, and A15 in Figure 13b,c,d, respectively), the same reaction mechanism as the U00 sample is observed during charging to 4.0 V, before the onset of the phase transition. The *c* lattice parameter of the P2 phase during the high-voltage phase transition on charge expands slightly from 11.3316(4) Å to 11.3416(5) Å for A05 and from 11.3254(4)­Å and to 11.3290(2) Å for A10, but shows a continual decrease for A15 from 11.3143(5) Å to 11.221(2) Å at the end of charge. This decrease in the *c* lattice parameter is accompanied by a decrease in the *a* lattice parameter to 2.830(15) Å at the end of charge. As a result, the unit cell volume further decreases to 77.83(8) Å^3^, which is 2.17% smaller than the volume that is typically maintained for the P2 phase (79.556(8) Å^3^ in U00). The evolving lattice parameters of the P2 phase during the P2–O2 phase transition shows the continuing deintercalation of Na^+^ ions from the P2 phase, which deviates from the classical two-phase reaction. This shows that Al-substitution can extend the solubility limit of Na in the P2 phase to *x* < 1/3. In a prior study by Pfeiffer et al. P2-type Na_*x*_[Ni_1/4_Mn_3/4_]­O_2_ showed a similar solid-solution reaction in the P2 phase at the end of charge with a decreasing *c* lattice parameter.^22^

The (002) reflection of the O2 phase emerges at 2.3°2θ (*d* = 4.49 Å) for A05, and 2.1°2θ (*d* = 4.92 Å) for A15. For the A10 sample the synchrotron operando cell exhibited an electrochemical capacity which was significantly lower than the typical coin cells, resulting in a negligible XRD signal from the O2 phase at the charged state. This O2 phase was observed with greater clarity from a laboratory operando XRD measurement (Figure S20), which corresponds to *d* = 4.76 Å of (002) for A10. This demonstrates an increase in the *c* lattice of the O2 phase with increasing Al-substitution (This trend is illustrated in Figure S23). This increasing *c* lattice of the charged phase effectively reduces the lattice mismatch between the P2 and O2 phases during the phase transition and has been attributed to the pillaring effect of trapped Na^+^ in the O2 phase that is necessary to maintain charge balance due to Al-substitution. While the (002) reflection of the O2 phase is sharp for U00 there is a large broadening for A05 and A10, and a slight broadening for A15 (Figures 13 and S20). This may be caused by a smaller average grain size or an increase of nonperiodic P2/O2 layer intergrowth caused by trapped Na^+^. At the fully charged state, aliovalent-substituted NaNMA has a theoretical composition of Na_*y*_^+^[Ni_1/3 *y*_^4+^Mn_2/3_^4+^Al_*y*_^3+^]­O_2_ with *y* = 0 for U00 or *y* = 0.05 for A05, so more Na^+^ ions are expected to be trapped in the O2 phase with increasing y. Komaba et al. examined Ti–Zn-substituted NaNM and found that the Na/vacancy superlattice was present at the discharged state, yet the high-voltage reaction was drastically altered to a pure solid solution mechanism.^45^ For this reason it is hypothesized that this trapped Na content in the charged (y Na per f.u.) phase alters the phase transition, rather than the disruption of Na/vacancy ordering in the discharged (1/3 Na per f.u.) phase.

Operando XRD for the isovalent-substituted NaNMA is shown in Figure 13e,f. These data were measured during the second half of the beamtime when the X-ray beam intensity was restored, so the data quality was sufficient to model the P2 phase with an orthorhombic unit cell. (Unfortunately, the data for U00 and the aliovalent-substituted samples do not have a sufficiently high signal-to-noise ratio to model the lattice distortion.) Both I06 and I11 samples exhibited significant lattice distortion, seen as a splitting between the two in-layer lattice spacings *a*′ and *b*′. The monoclinic angle, γ′, for I06 decreased from 120.120(3)° at the start of charge (from refined results based on the as-synthesized sample in Figure 6) to a negligible distortion early during charging (Figure S22 for expanded view) and then increased to 121.091(2)° at the highly charged state at 4.0 V (first observed once the beam intensity was restored). Since the initial charging was done during the low beam intensity, the presence or absence of the distortion at Na_1/3_ in Na_*x*_[Ni_1/3‑*y*/2_Mn_2/3‑*y*/2_Al_*y*_]­O_2_ was only observable during discharging when the signal-to-noise ratio is sufficiently high. The distortion had a continuous shift during discharging from 121.091(2)° at 3.95 V to a negligible 120.001(4)° at 3.66 V, which shows a greater distortion for the charged P2 phase above 3.95 V than the as-synthesized phase. Similar observations were made for the I11 sample. This lattice distortion is attributed to cooperative Jahn–Teller distortion of Ni^3+^, whose concentration increases from *x* = 0 (at 2.8 V) to *x* = 0.30 (at 4.0 V) in Na_0.66‑*x*_[Ni_0.30‑*x*_^2+^Ni_*x*_^3+^Mn_0.66_^4+^Al_0.03_^3+^]­O_2_ (Ni, Mn, Al from combined NPD and XRD refinement of I11). The evolution of the lattice parameters for the I06 and I11 samples is similar to the U00 sample, where the *c* (*a*) lattice parameter increases (decreases) to 11.359(3) Å (2.843(2) Å) upon charging to 4.0 V and remains constant until the end of charge.

The (002) reflection for the O2 phase shows a bimodal profile, which is modeled by two phases referred to as “O2 Phase 1” and “O2 Phase 2”. Example fitting profiles are shown in Figure S21. Phase 1 with a *c* lattice parameter of 9.0 Å is similar to the typical O2 phase observed for Na_*x*_[Ni_1/3_Mn_2/3_]­O_2_, while the *c* lattice parameter at 9.25 to 9.35 Å for Phase 2 suggests an intergrowth of O and P-type layers.^61,62^ However, the *c* lattice parameter for Phase 2 is much smaller than the typical OP4 phase, where equal concentration of the P- and O-type layers is expected to yield an effective *c* lattice of 10.12(4) Å, which is an average of the *c* lattices for the P2 (*c* = 11.359(3) Å) and O2 (*c* = 8.876(3) Å) phases. This value is also within the range typically reported for OP4 phases in similar layered oxides (e.g., Na_2/3_[Ni_1/6_Mn_1/2_Fe_1/3_]­O_2_).^63^ Therefore, the smaller *c* lattice than what is typically observed for the OP4 phase suggests a greater concentration of O stacking layers in the present case. This result might be better understood under the structure model of Z phases with variable concentrations of O and P stacking, with Na_*x*_[Fe_1/2_Mn_1/2_]­O_2_ as a notable example.^61,63^ The peak from Phase 2 was most prominent during the first charge and decreased in intensity with subsequent cycles.

Since both the I06 and I11 samples have similar Al content in the layered phase to the A05 and A10 samples, the differences in the high-voltage P2–O2 phase transitions cannot be explained by the Al content alone. Therefore, the high-voltage phase transition mechanism is likely affected by the Ni/Mn ratio, too. The P2 phase behavior in A15 is similar to the reaction seen in the Mn-rich Na_*x*_[Ni_1/4_Mn_3/4_]­O_2_ with a collapsing *c* lattice parameter above 4.2 V which supports this conclusion.^22^

## 4 Conclusions

NaNMA was investigated to determine the substitution of Al into the bulk structure via the aliovalent and isovalent substitution strategies. Analysis of both the XRD and NPD results confirms Al-substitution in the layered P2 phase, which affects Na^+^-vacancy ordering and the lattice distortion. It was determined that the aliovalent substitution strategy allows a higher concentration of Al in the layered phase than the isovalent substitution strategy under identical synthesis conditions. Regardless of the substitution strategy, Al preferentially substitutes Ni and occupies the Ni site. Such a preference is likely present for substituting with other elements and should be considered when designing substitution strategies.

The difference in actual content of Al in the layered phase accounts for the major differences in the electrochemistry of samples prepared by both substitution methods. In the aliovalent substitution strategy, there is a gradual improvement in capacity retention as the Al content increases until A07 with an Al content from combined refinement of 0.060(5) per f.u. Aliovalent-substituted samples with greater ICP Al content have only the detriment of a lower initial capacity without an increasing capacity retention. All of the isovalent-substituted samples had *ca*. 0.036 Al content from combined refinement, which resulted in similar but improved capacity retention between the isovalent-substituted samples.

Significant differences in the phase evolution between samples prepared by different substitution strategies are revealed by operando XRD. The aliovalent-substituted samples, with the co-occupation of both Al and Mn at the Ni site, show an expanded Na solubility limit in the P2 phase and a reduced volume difference between the P2 and O2 phases. This increasing average *c* lattice of the O2 phases combined with the broadened O2 peak shapes suggest homogeneous yet disordered intergrowth of P2 and O2 layers at the charged state. In contrast, the isovalent-substituted samples, with the co-occupation of only Al at the Ni site, show a heterogeneous O–P intergrowth phase during the high-voltage phase transition. Our result demonstrates the importance of understanding mixed metal occupancies in designing elemental substitution strategies for Na-ion battery cathode materials.

## Supplementary Material



## Data Availability

All XRD and NPD data, the TOPAS Academic scripts used to analyze them, XANES, electrochemical, and ICP-OES data, are available on Zenodo at doi.org/10.5281/zenodo.17308427.

## Notes

Additional analyses of the powder diffraction and electrochemistry. Tables for elemental analysis, refined structure parameters, and chemical formulas (PDF)

## Abbreviations

LIB: Li-ion battery
LTMO: layered transition metal oxide
NaNM: P2-type Na_2/3_[Ni_1/3_Mn_2/3_]­O_2_
NaNMA: P2-type Na_2/3_[Ni_*x*_Mn_*y*_Al_*z*_]­O_2_ (*x* + *y* + *z* = 1)
NIB: Na-ion battery
NPD: neutron powder diffraction
S.G.: space group
TM: transition metal
XANES: X-ray absorption near-edge spectroscopy
XRD: X-ray diffraction
