# Supplementary material for: Structure, Electrochemistry, and Phase Evolution of Al-Substituted Na2/3[Ni1/3‑yMn2/3‑zAl y+z ]O2 as a Sodium-Ion Battery Cathode Material
Source: Chem Mater. 2026 May 19;38(11):5369–83. doi: 10.1021/acs.chemmater.5c03184 (PMC13289818; doi:10.1021/acs.chemmater.5c03184)
Supplement: Supplementary file 1 [file cm5c03184_si_001.pdf]

Supporting Information

Structure, electrochemistry, and phase evolution of Al-substituted $Na_{2/3}[Ni_{1/3-y}Mn_{2/3-z}Al_{y+z}]O_2$ as a Sodium-Ion Battery Cathode Material

Anthony T. Pacileo[1], *Patrick Deegan*[1,2], *Hao Liu*[1,2]*

[1] Materials Science and Engineering Program, Binghamton University, Binghamton, New York 13902, United States

[2] Department of Chemistry, Binghamton University, Binghamton, New York 13902, United States

Statement on the Structure Solution

Several structure models have been proposed for the Na-vacancy ordering in $Na_{2/3}[Ni_{1/3}Mn_{2/3}]O_2$. While these models could account for the position of the superlattice reflections, our fitting results using existing models show poor agreement with the observed peak intensities. Meng et al[1] discovered arrangements of Na from density functional theory, but this model produces extra reflections which are not observed. The model from Komaba et al[2] matches many of the measured intensities but the Na site coordinate was refined, which moves the Na off the high symmetry position, resulting in multiple Na-O bond lengths.

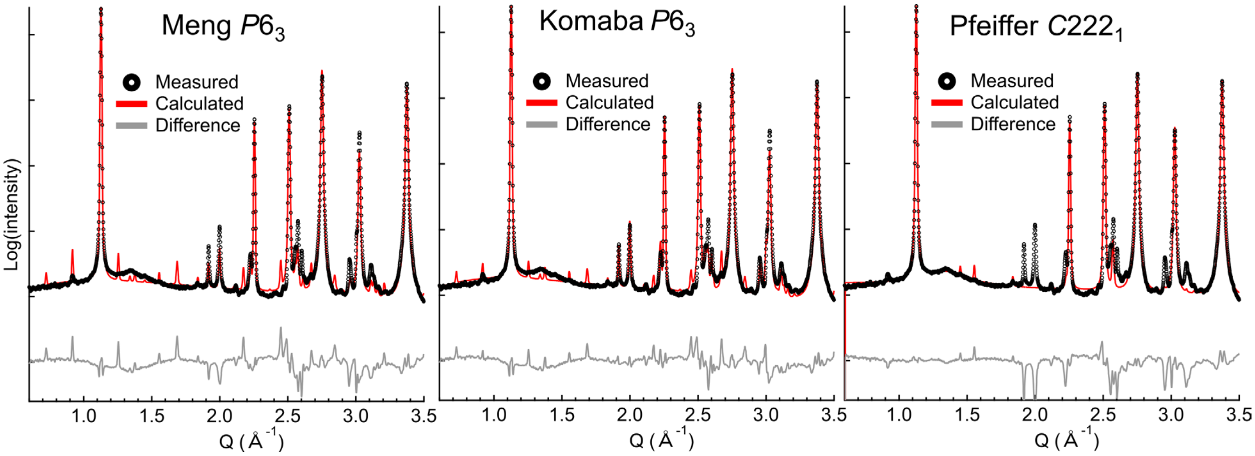

Figure S1 . *Fitting profiles for published structure models from Meng*[1]*, Komaba*[2]*, and Pfeiffer* [3].

We have done indexing of the powder patterns to search for space groups which might produce these patterns. However, none of the top indexing results are compatible with the “AB honeycomb” transition metal arrangement proposed by Pfeiffer *et al.* [3] The combined requirements for modelling Ni/Mn ordering and Na-vacancy ordering make this structure solution significantly more difficult than finding models for the individual ordering alone (i.e., Ni/Mn ordering or Na/vacancy ordering alone). High intensity single crystal X-ray or electron diffraction will greatly aid in peak indexing, so we are planning for further single crystal diffraction experiments to provide additional experimental input in peak indexing. Meanwhile, the XRD and NPD source files are publicly available in the electronic supporting information to encourage the community to make progress toward the structure solution.

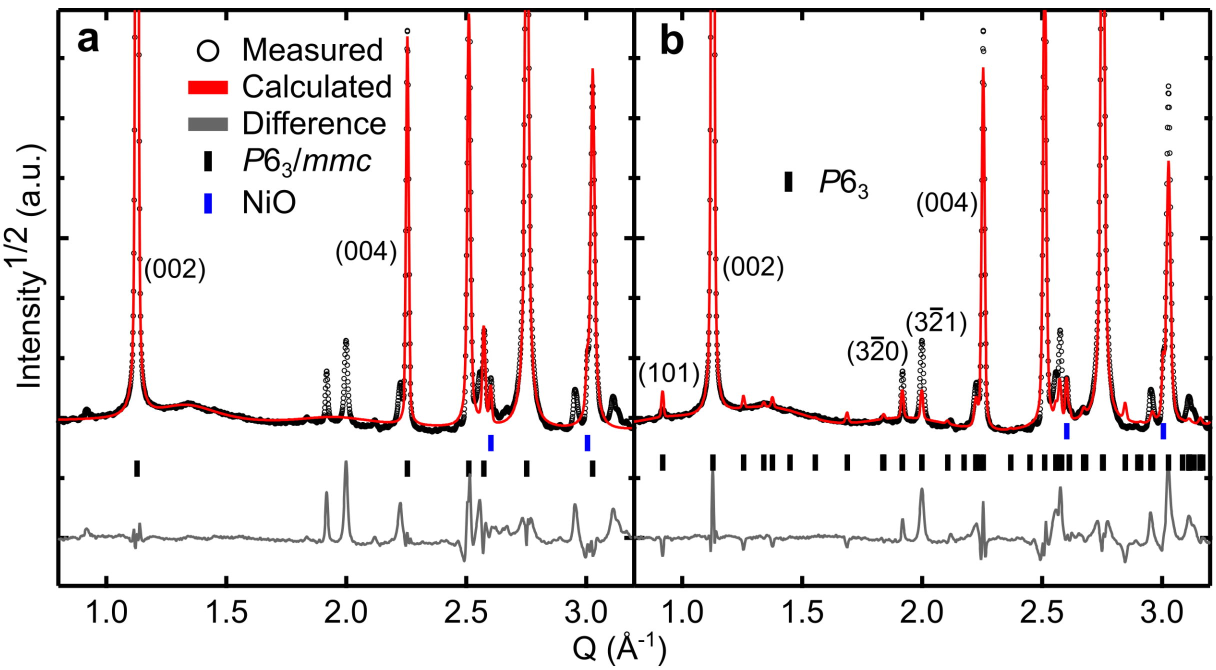

Figure S2. Rietveld refinement results for synchrotron XRD on $Na_{2/3}[Ni_{1/3}Mn_{2/3}]O_2$ using (**a**) the small $P6_3/mmc$ unit cell which does not account for Na-vacancy ordering. The pattern is approximately fit with (**b**) the large $2\sqrt{3}a$ $P6_3$ unit cell which indexes the reflections at Q = 0.9, 1.8, 1.9, 2.0, and 2.2 Å^{-1}, however the intensities are not accurate following Rietveld refinement of the Na^+ occupancy at the 10 unique Na^+ sites. The $(3\bar{2}0)$ and $(3\bar{2}1)$ reflections are indexed as $(1\bar{2}0)$ and $(1\bar{2}1)$ in the orthorhombic unit cell with S.G. $C222_1$, however, these are forbidden in $C222_1$.

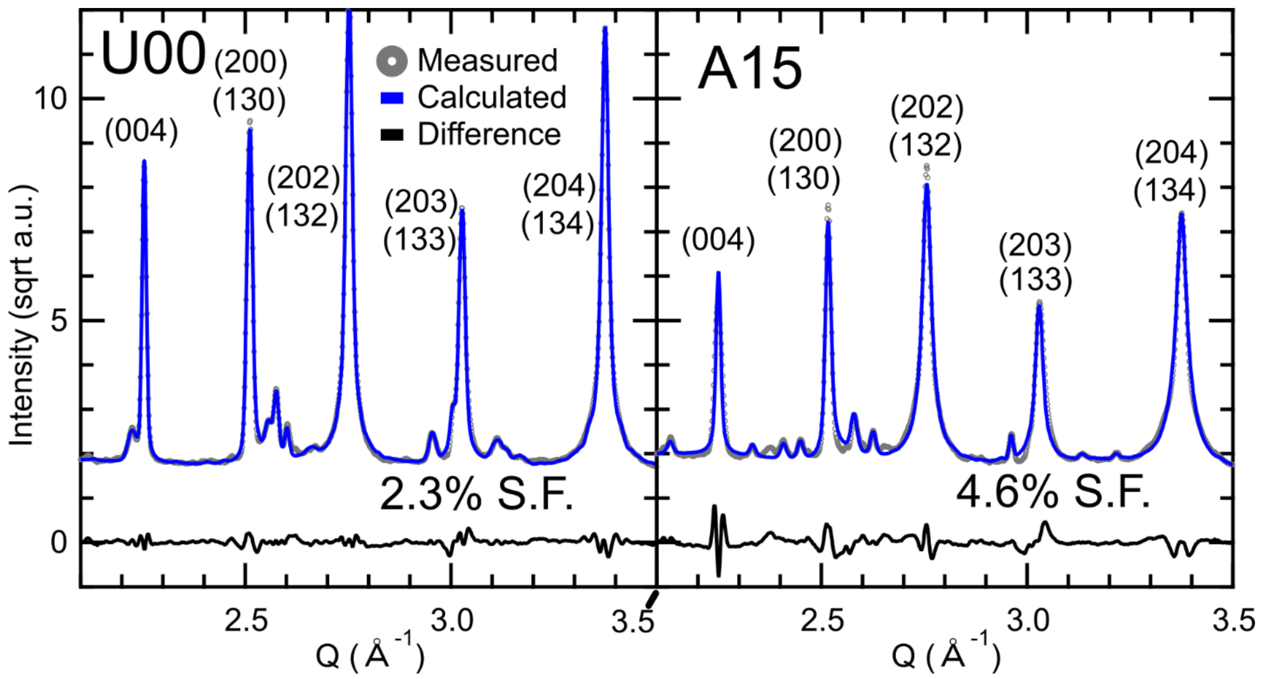

Figure S3. XRD Rietveld fitting for $Na_{2/3}[Ni_{1/3}Mn_{2/3}]O_2$ (U00) and the aliovalent ICP $Al_{0.15}$ (A15) samples with insertions of P3 stacking faults (S.F.). These S.F. induce anisotropic Lorentzian broadening on the (13*l*) and (20*l*) with $l > 0$ class of reflections, while (hk0) and (00*l*). remain relatively sharp.

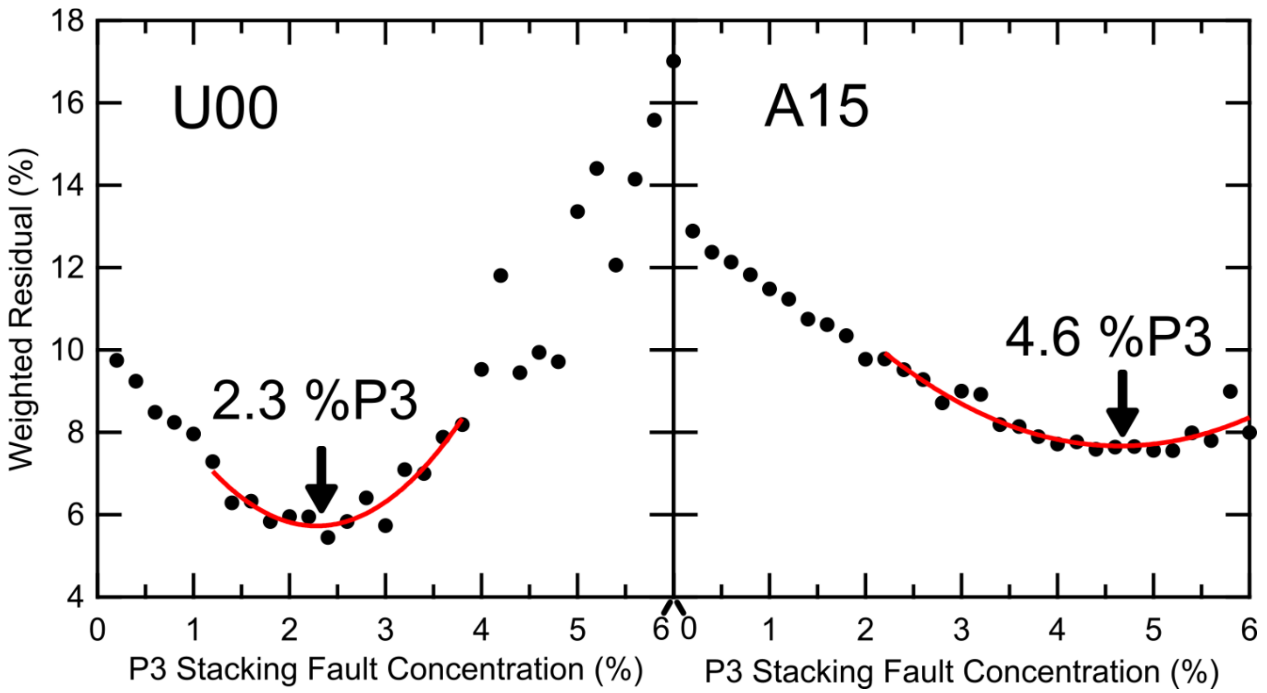

Figure S4. Results from XRD Rietveld fitting of U00 and A15 with P3 layer insertions. TOPAS-Academic cannot change the simulated stack of atomic layers during refinement. To explore a range of values from 0 to 6 % P3 layer insertions in the P2 structure, these stacking sequences were simulated sequentially. A parabola was fitted to a subrange of the stacking fault concentration vs R_{wp} data to determine the stacking fault concentration corresponding to lowest R_{wp}.

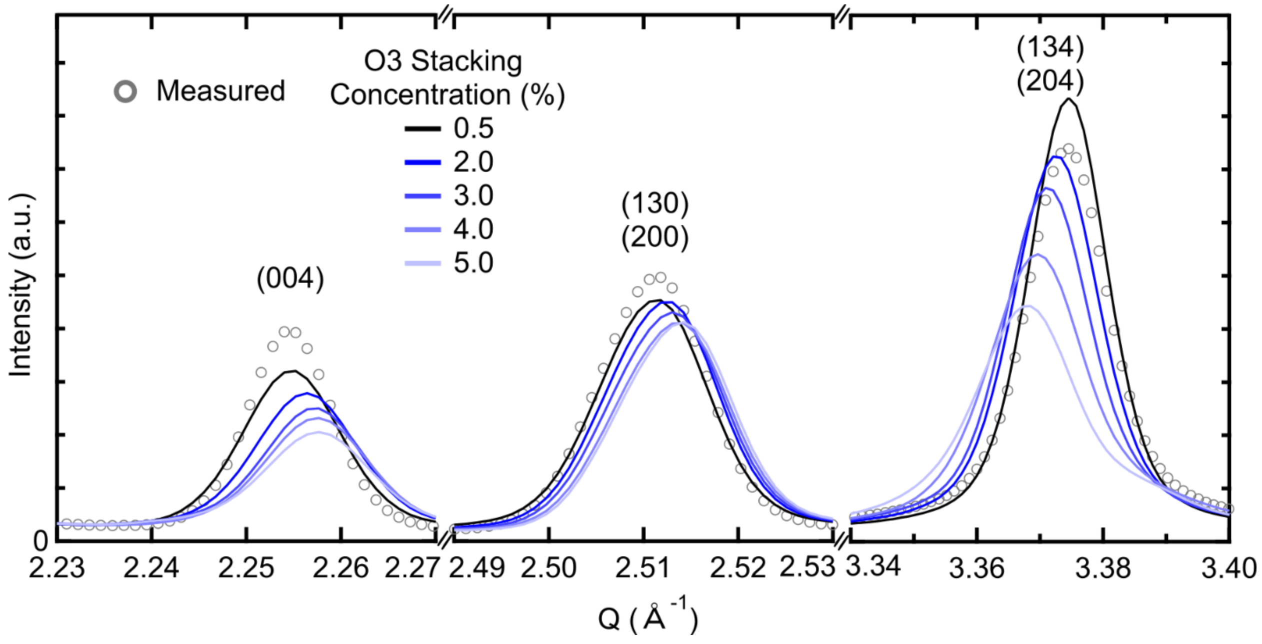

Figure S5. Calculated XRD patterns for the P2 structure with O3 layer insertions using an orthorhombic unit cell. The O3 interlayer distance was set to 5.31833 Å from Komaba *et al.*[4] and the P2 interlayer distance to 5.6 Å. The *a*, *b*, and average *c* lattice parameters for the stacking sequences were refined. The skewed peak positions demonstrate that stacking faults in these samples must not involve a decreased interslab distance such as O2 or O3 stacking, instead pointing to insertions of P3 stacking which have the same interslab distance as P2.

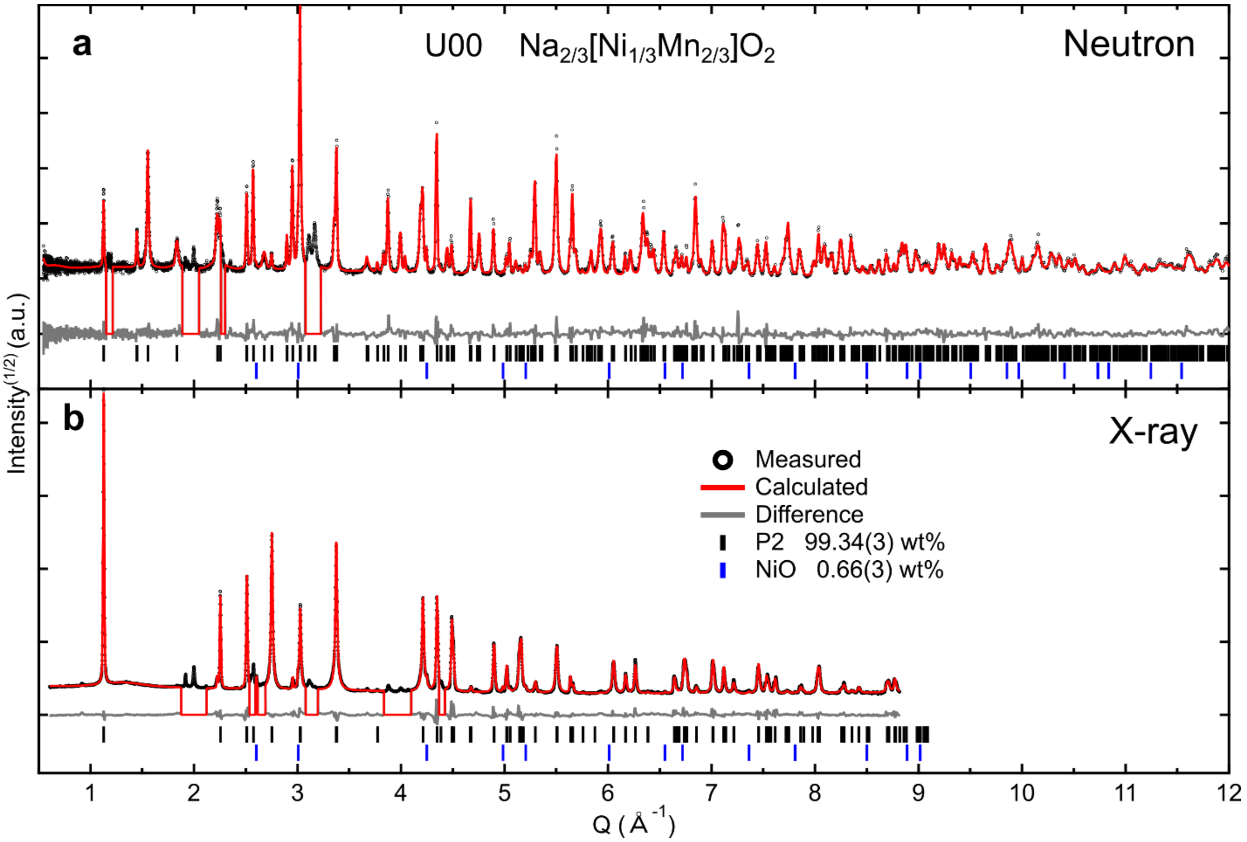

Figure S6. Combined (**a**) NPD and (**b**) XRD Rietveld refinement for U00 (nominal composition $Na_{2/3}[Ni_{1/3}Mn_{2/3}]O_2$).

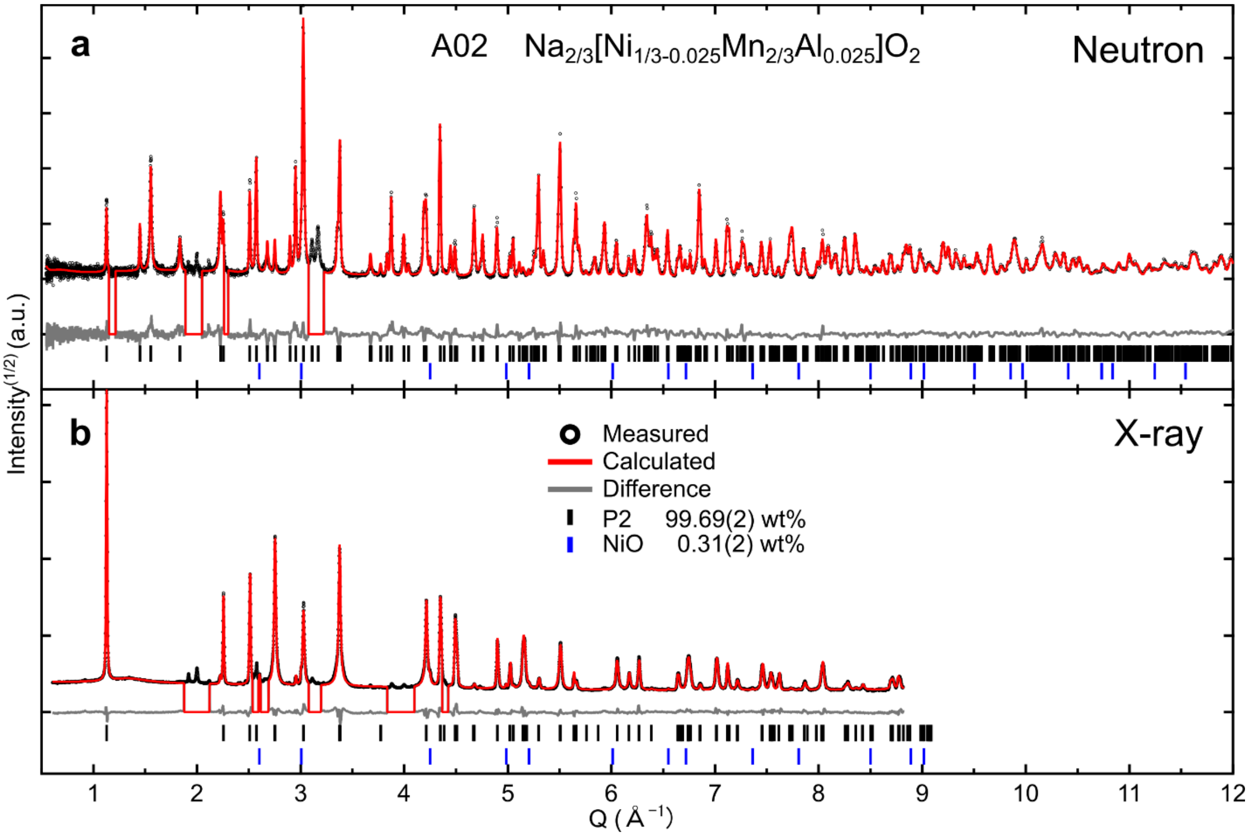

Figure S7. Combined (**a**) NPD and (**b**) XRD Rietveld refinement for A02 (nominal composition $Na_{2/3}[Ni_{1/3-0.025}Mn_{2/3}Al_{0.025}]O_2$)

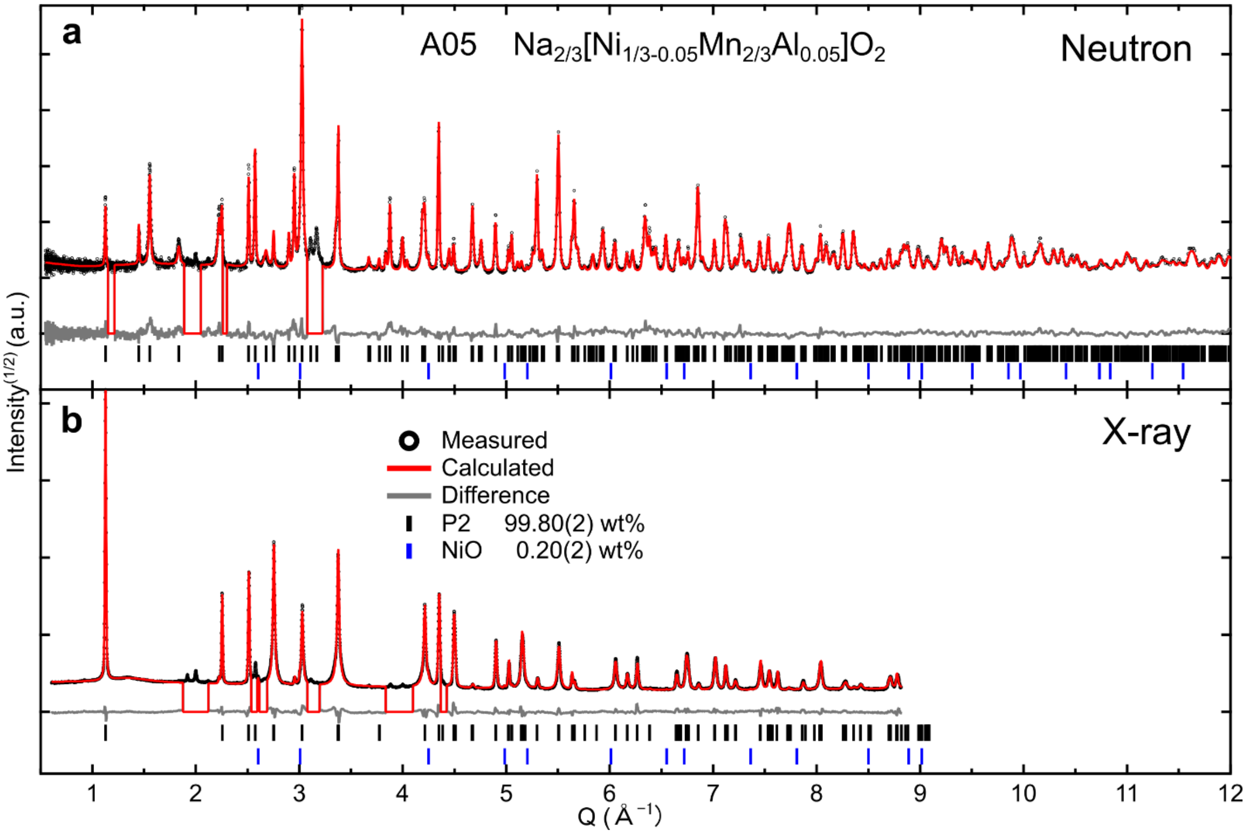

Figure S8. Combined (**a**) NPD and (**b**) XRD Rietveld refinement for A05 (nominal composition $Na_{2/3}[Ni_{1/3-0.05}Mn_{2/3}Al_{0.05}]O_2$).

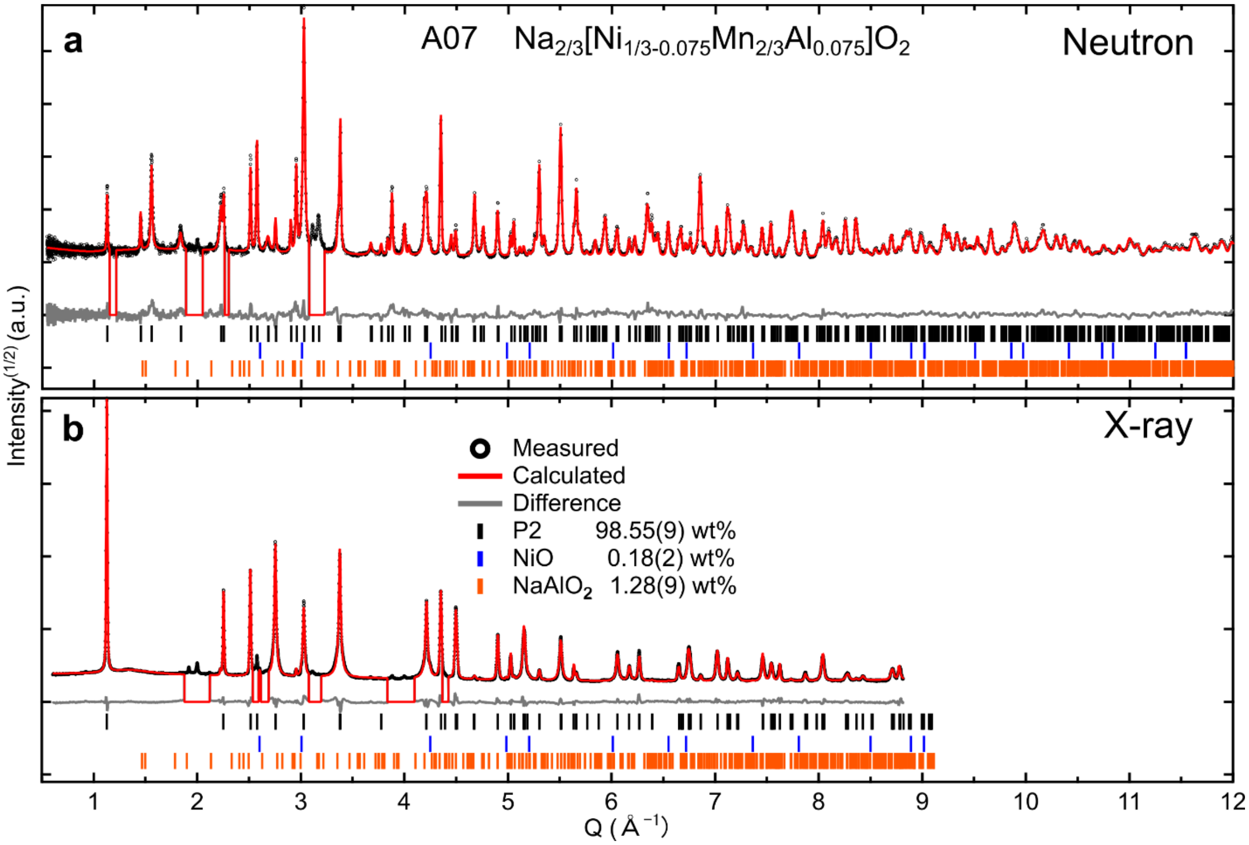

Figure S9. Combined (**a**) NPD and (**b**) XRD Rietveld refinement for A07 (nominal composition $Na_{2/3}[Ni_{1/3-0.075}Mn_{2/3}Al_{0.075}]O_2$).

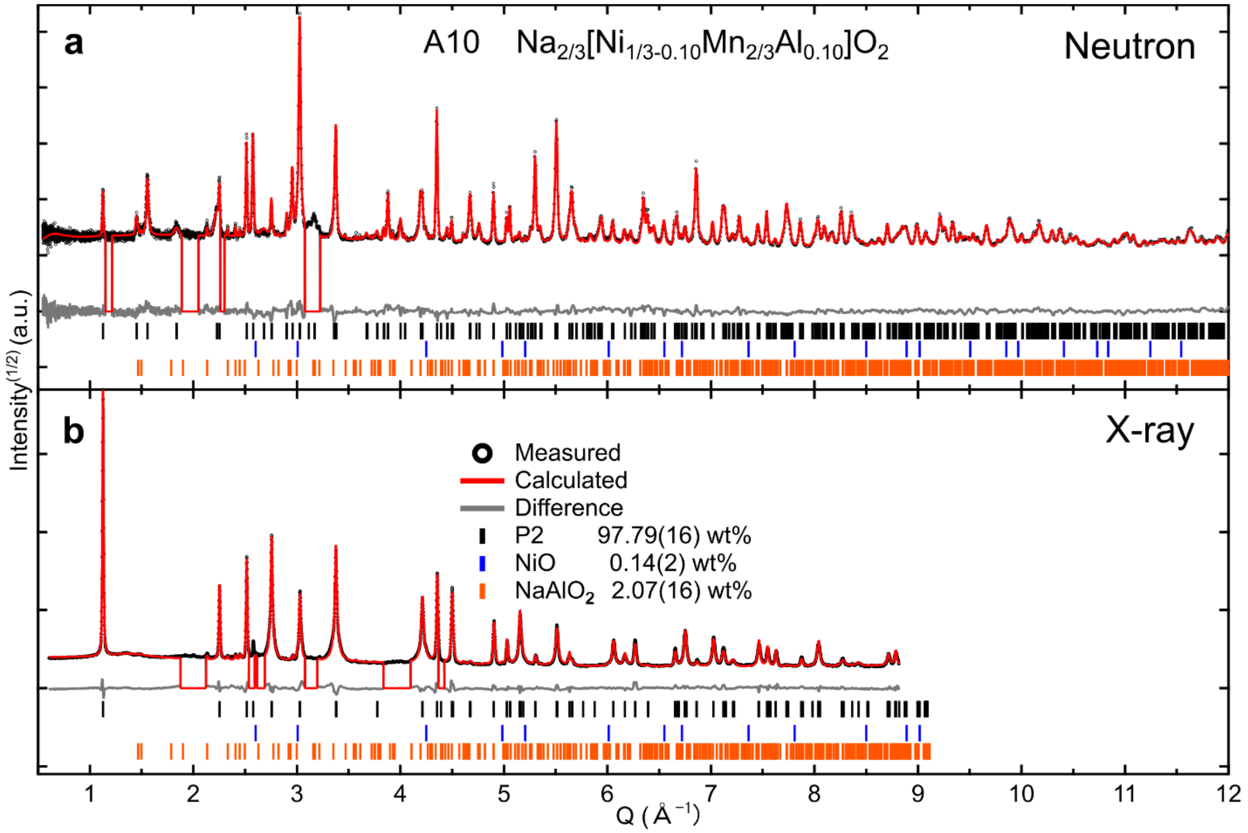

Figure S10. Combined (**a**) NPD and (**b**) XRD Rietveld refinement for A10 (nominal composition $Na_{2/3}[Ni_{1/3-0.10}Mn_{2/3}Al_{0.10}]O_2$).

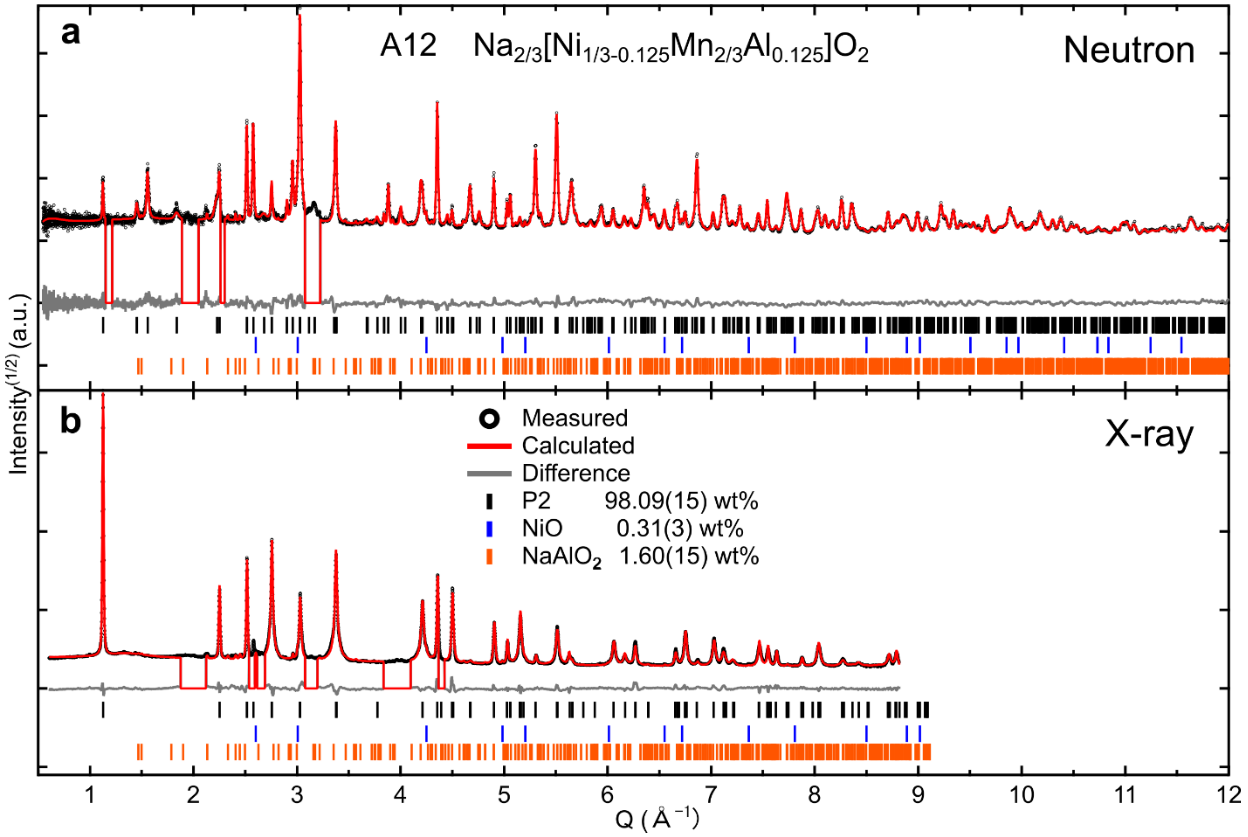

Figure S11. Combined (**a**) NPD and (**b**) XRD Rietveld refinement for A12 (nominal composition $Na_{2/3}[Ni_{1/3-0.125}Mn_{2/3}Al_{0.125}]O_2$).

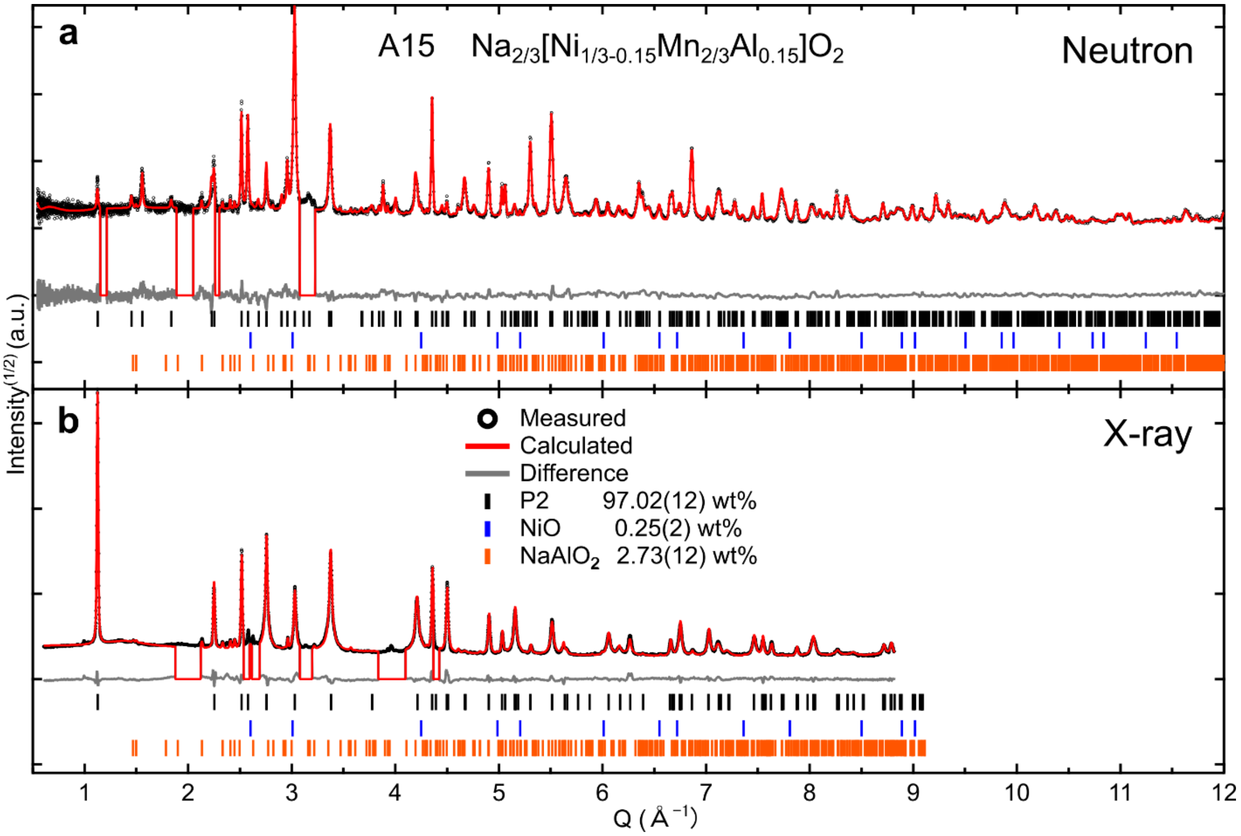

Figure S12. Combined (**a**) NPD and (**b**) XRD Rietveld refinement for A15 (nominal composition $Na_{2/3}[Ni_{1/3-0.15}Mn_{2/3}Al_{0.15}]O_2$).

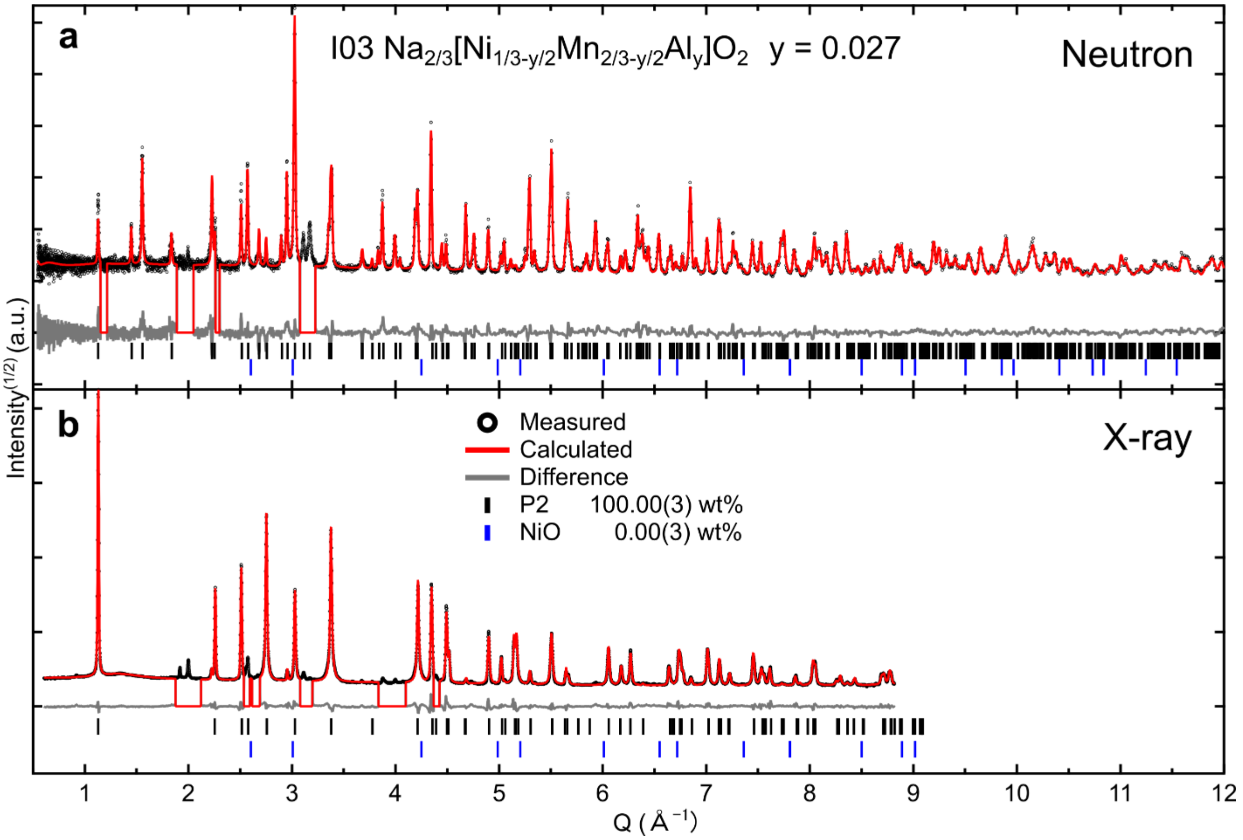

Figure S13. Combined (**a**) NPD and (**b**) XRD Rietveld refinement for I03 (nominal composition $Na_{2/3}[Ni_{1/3-(0.027/2)}Mn_{2/3-(0.027/2)}Al_{0.027}]O_2$).

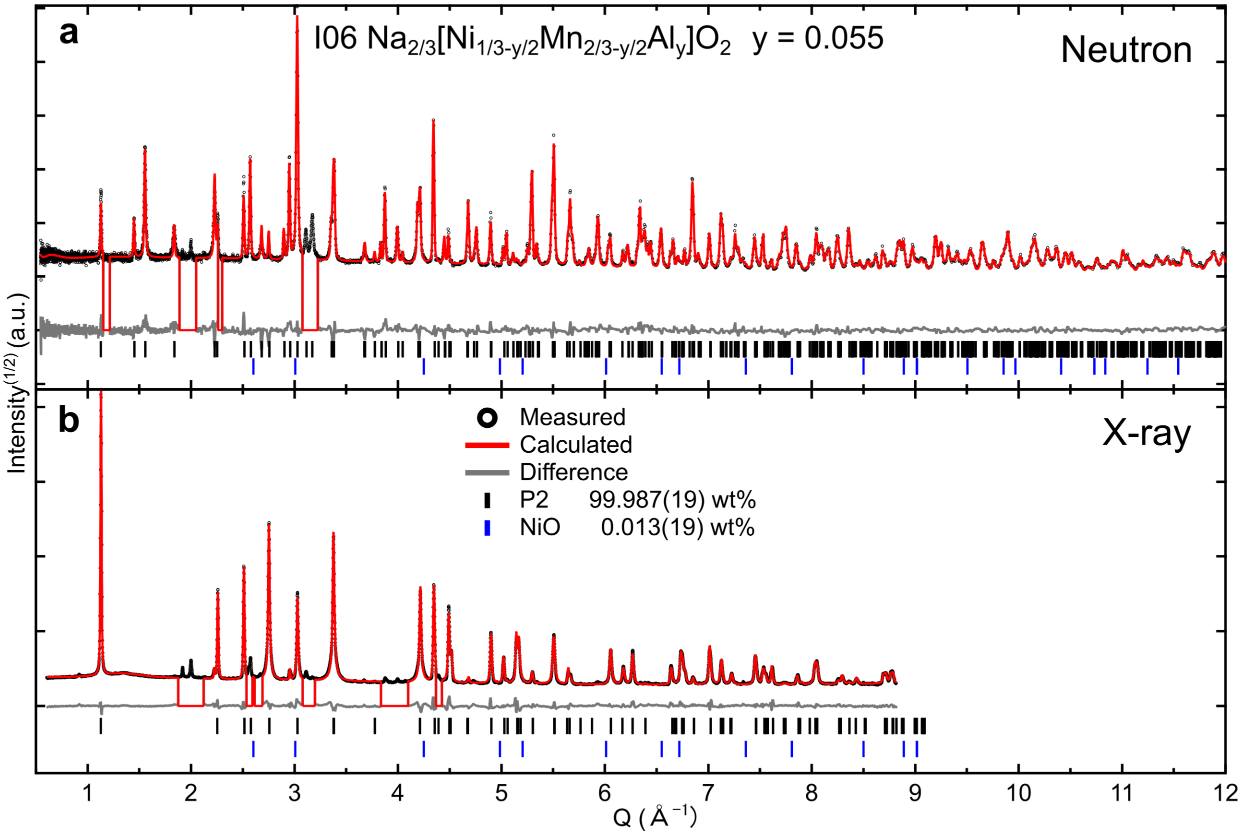

Figure S14. Combined (**a**) NPD and (**b**) XRD Rietveld refinement for I06 (nominal composition $Na_{2/3}[Ni_{1/3-(0.055/2)}Mn_{2/3-(0.055/2)}Al_{0.055}]O_2$).

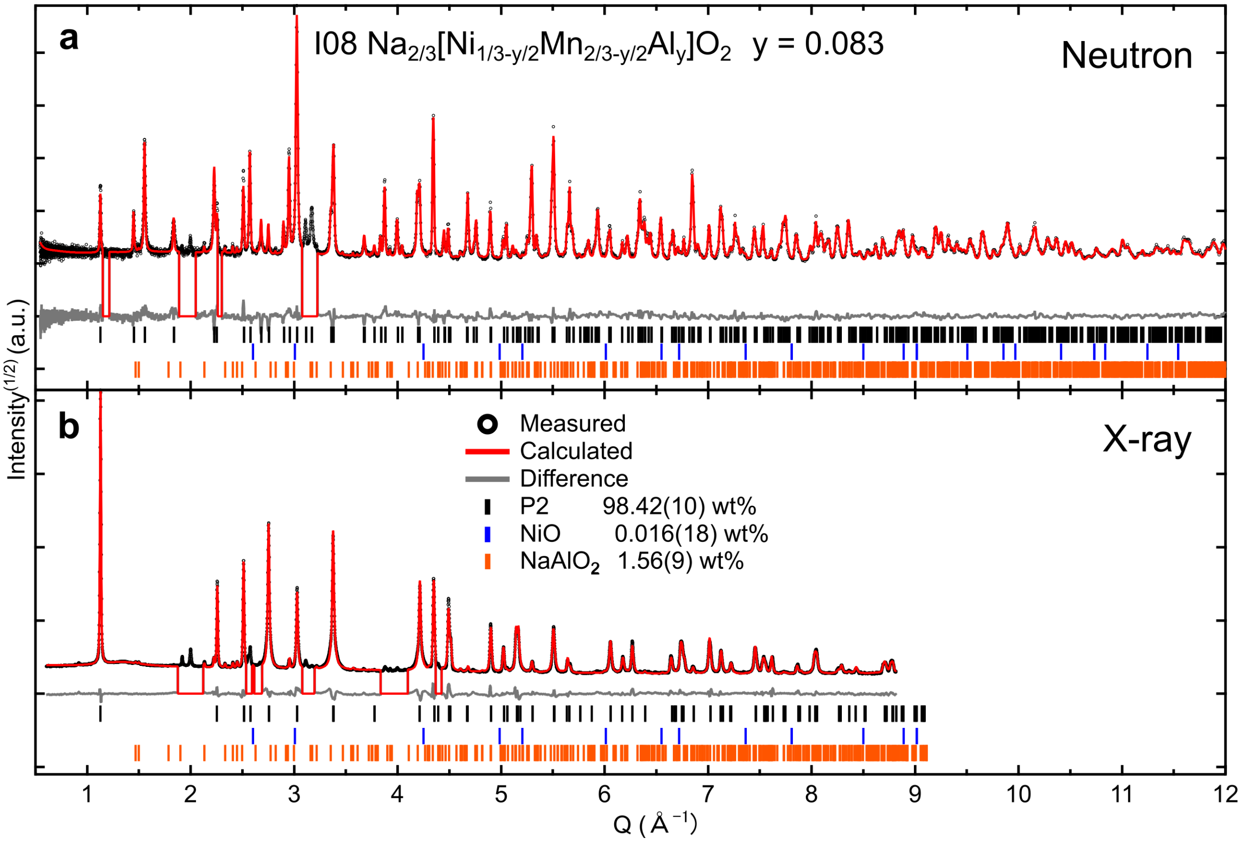

Figure S15. Combined (**a**) NPD and (**b**) XRD Rietveld refinement for I08 (nominal composition $Na_{2/3}[Ni_{1/3-(0.083/2)}Mn_{2/3-(0.083/2)}Al_{0.083}]O_2$).

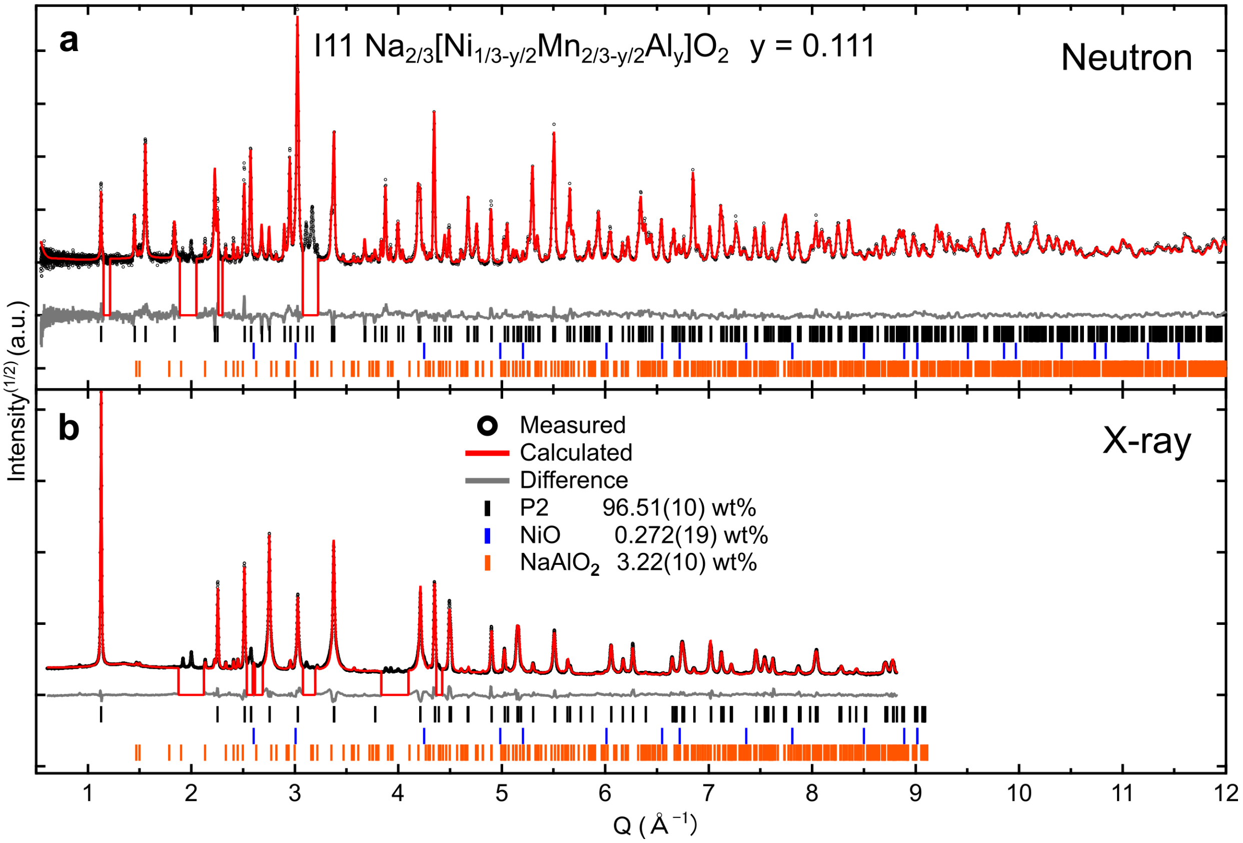

Figure S16. Combined (**a**) NPD and (**b**) XRD Rietveld refinement for I11 (nominal composition $Na_{2/3}[Ni_{1/3-(0.11/2)}Mn_{2/3-(0.11/2)}Al_{0.11}]O_2$).

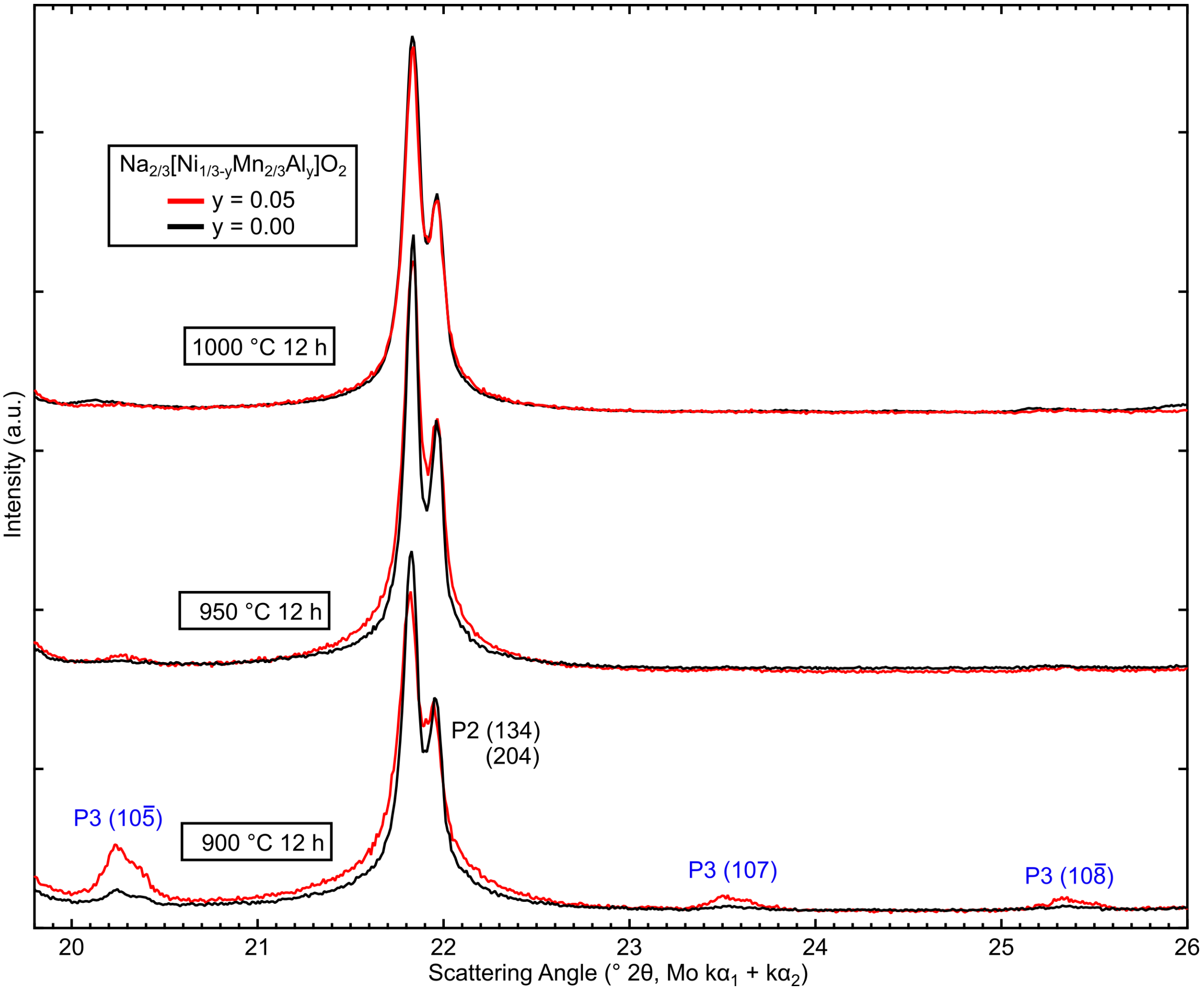

Figure S17. XRD patterns of NaNMA with increasing solid state synthesis temperature collected on a Bruker D8. Double peaks are caused by the dichromatic X-ray beam. The P2 reflections are indexed *via* space group $C222_1$. The samples produced at 900 °C have characteristic peaks from the P3 $Na_{2/3}[Ni_{1/3}Mn_{2/3}]O_2$ phase with space group $R3m$.[5] At 900 °C the aliovalent-substituted sample with a nominal composition of y = 0.05 in $Na_{2/3}[Ni_{1/3-y}Mn_{2/3}Al_y]O_2$ has a significantly higher concentration of this P3 phase *vs* the unsubstituted y = 0.00 sample. The y = 0.05 at 900 °C sample also has a large broadening of the P2 (134) and (204) reflections, indicative of P3-intergrowth-type stacking faults. Increasing the synthesis temperature to 950 °C sharpens these P2 reflections and nearly eliminates the P3 phase. At 1000 °C the P3 phase is no longer detected and the P2 phase has sharper (134) and (204) reflections, indicating the P3 stacking content in the P2 phase is significantly reduced. This trend of increasing temperature for the P2/P3 phase boundary with increasing Al content in NaNMA was also observed by Kumar *et al.*[6]

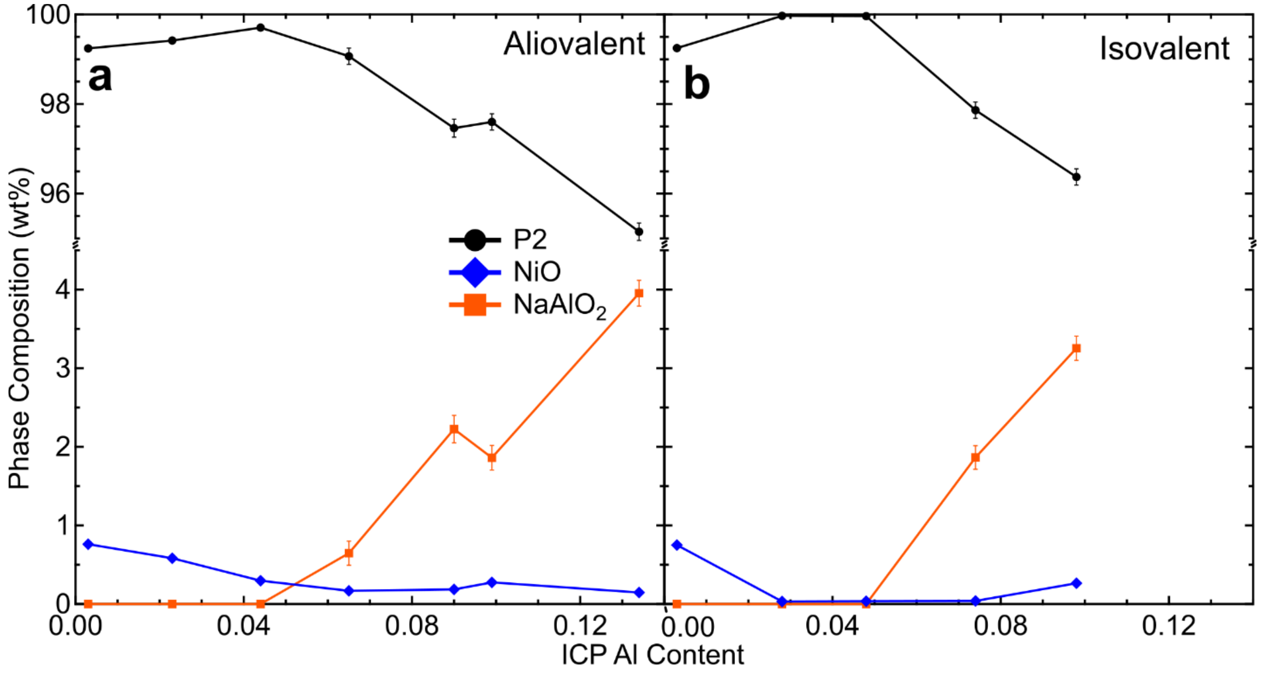

Figure S18. Phase concentrations measured by Rietveld refinement of synchrotron XRD for (**a**) aliovalent- and (**b**) isovalent-substituted $Na[NiMnAl]O_2$. The layered NaTMO phase is designated as P2 and shown on a split axis for clarity.

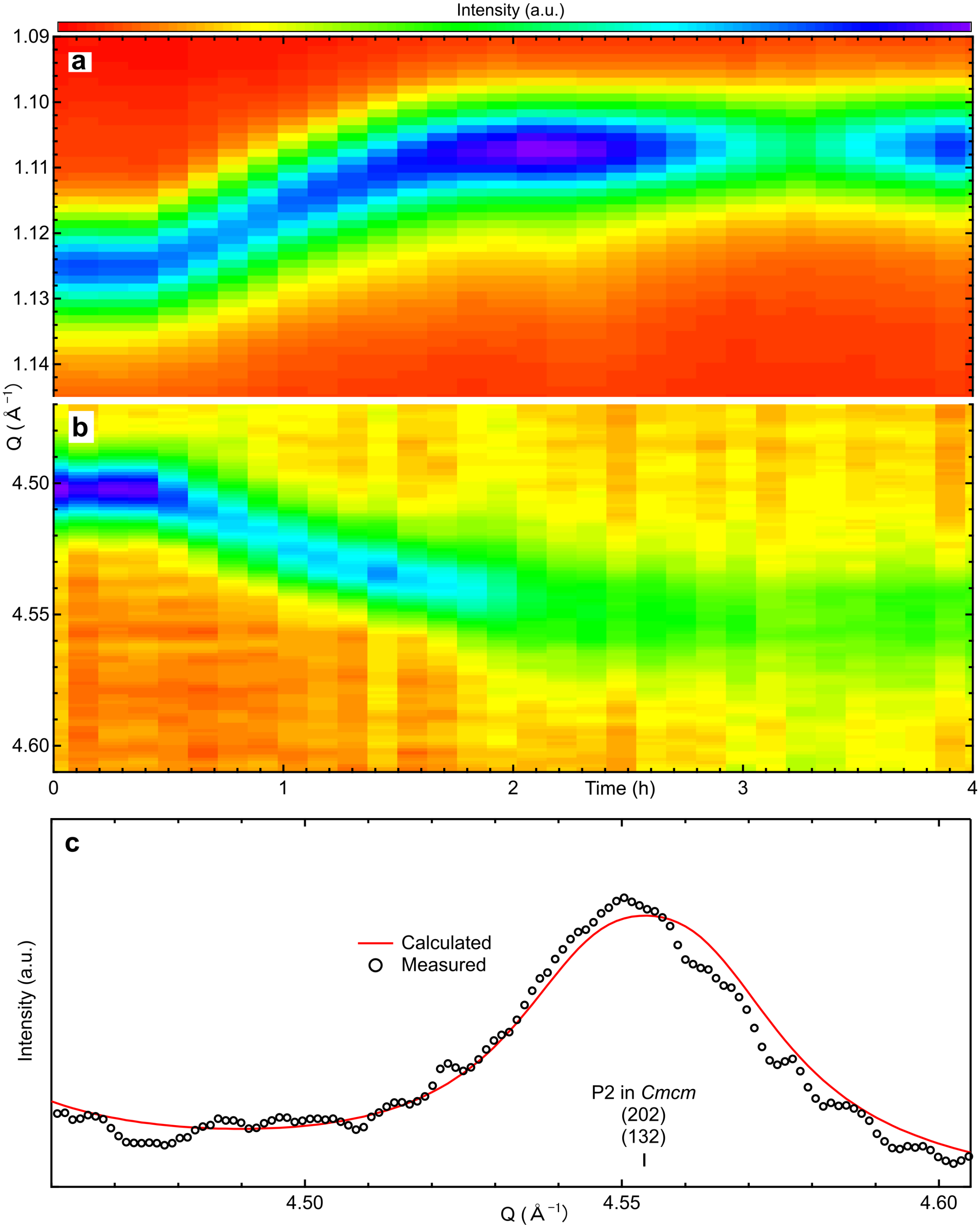

Figure S19. Operando XRD for A05 with the P2 (**a**) (002) and (**b**) (202) and (132) reflections indexed *via* the *Cmcm* space group. (**c**) Profile fitting at 2.08 h (charged to 1/3 Na content per f.u.) shows no splitting of these reflections which would indicate an orthorhombic distortion, and that a hexagonal model is appropriate. See **Figure S22a** and **Figure S22b** for comparison as distortion is observed in this Q region for I06 and I11.

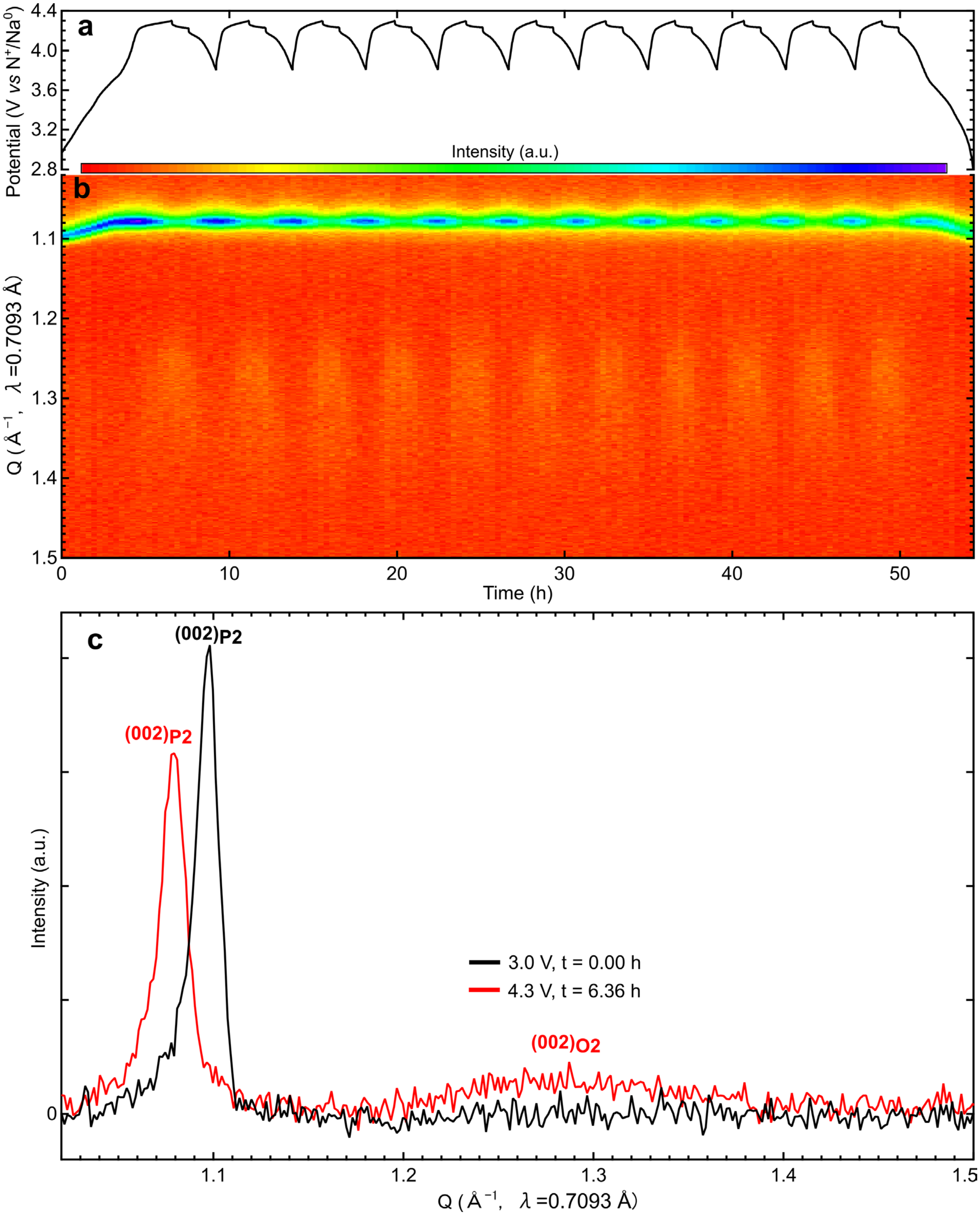

Figure S20. Operando XRD for sample A10 collected on a Bruker D8 with a Mo X-ray source. (**a**) voltage profile, (**b**) false-color view of XRD patterns in P2 and O2 (002) region, and (**c**) select XRD patterns showing the appearance of a peak from the desodiated phase (*e.g.* O2, OP4 or Z). The very broad shape of the desodiated phase peaks and low signal-to-noise prohibits phase identification.

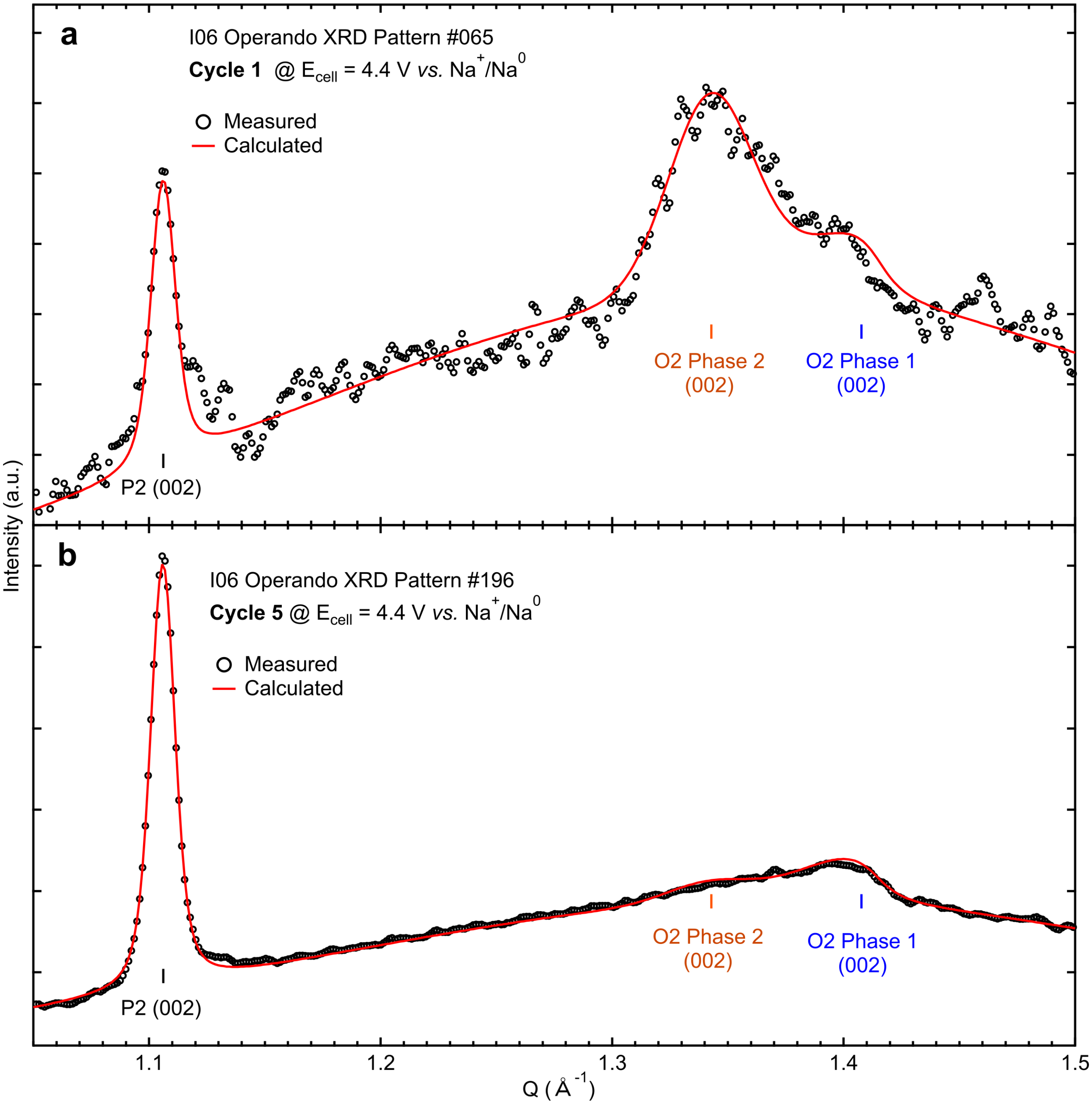

Figure S21. Operando XRD patterns from I06 at the fully charged 4.4 V state during the (**a**) 1st and (**b**) 5th charge-discharge cycles. At the end of the initial charging there was a relatively large amount of "O2 Phase 2" with (002) at lower Q compared with "O2 Phase 1" (002) at higher Q. In the subsequent charge/discharge cycles the peak from "O2 Phase 2" has a decreased intensity at the 4.4 V state.

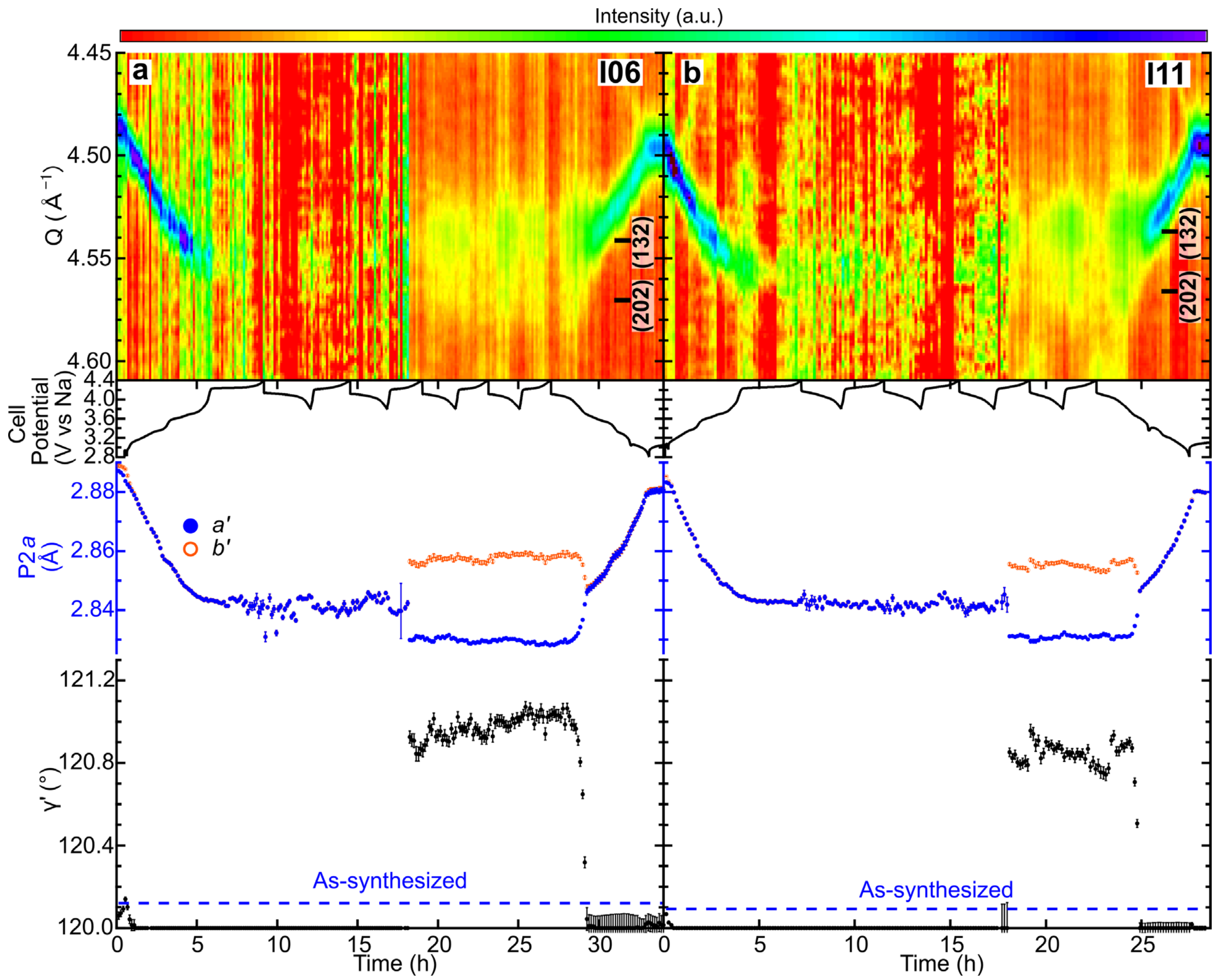

Figure S22. Expanded view of the γ', *a*', and *b*' from operando XRD on (**a**) I06 and (**b**) I11 showing reflections from the P2 phase which are indicative of orthorhombic distortion. These are indexed as (132) and (202) in space group *Cmcm*. These XRD patterns were used to refine the pseudo-hexagonal *a*' and *b*' which yield an equivalent monoclinic angle γ'. The γ' of the as-synthesized materials are shown for comparison. This initial distortion is relaxed within the first 3-5 scans during charging and does not reappear at the end of discharging as the materials do not reach the same state of discharge. The γ' angle is fixed to 120 ° from 1.3 to 18 h as the XRD patterns have too low signal/noise ratio to measure the high-Q reflections, especially (132) and (202). Above 4.0 V the P2 material has a nearly constant distortion, first visible after 18 h once the synchrotron beam intensity was restored. As they are discharged to 3.6 V the distortion is eliminated and does not reappear at the end of discharge, as they do not achieve the same state of discharge as the as-synthesized samples.

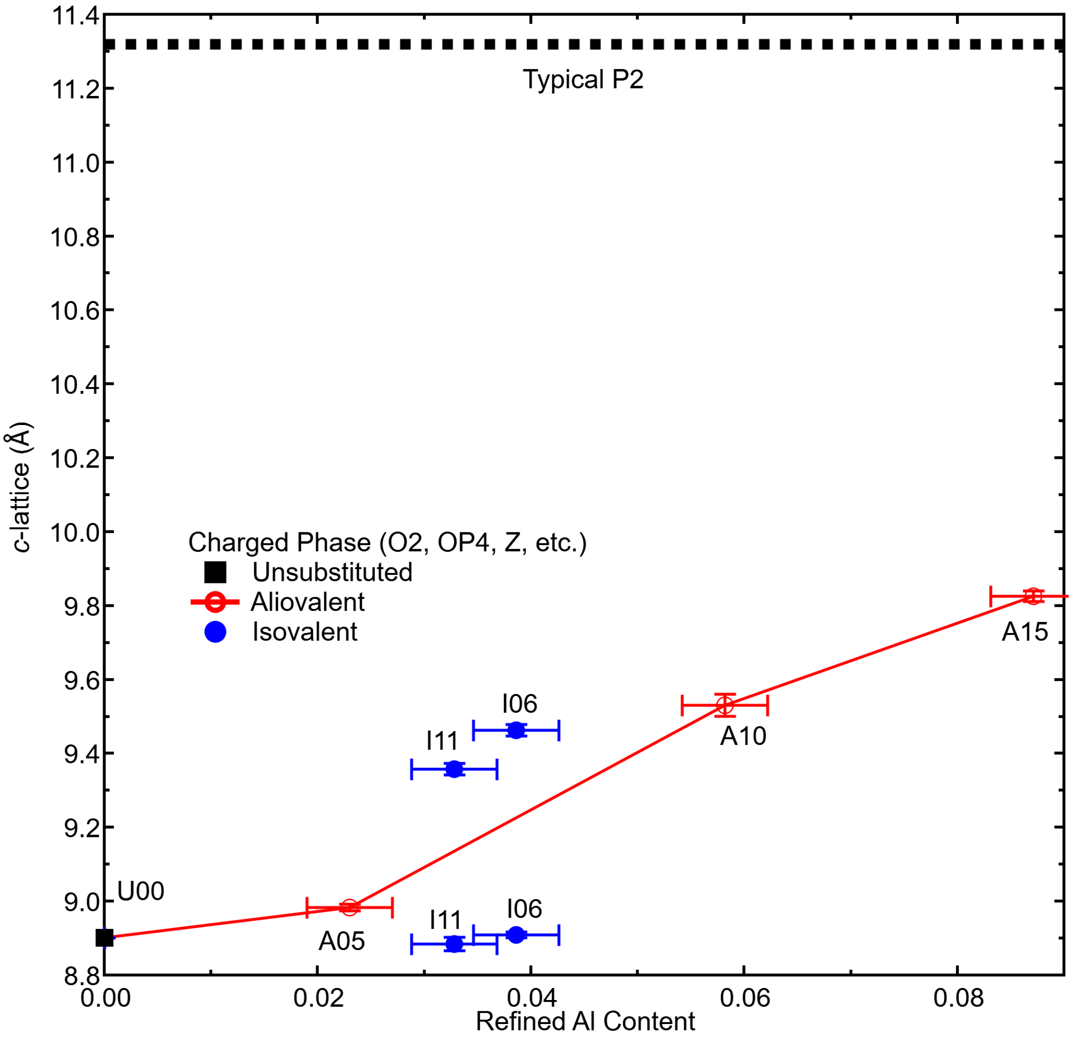

Figure S23. Charged phase *c*-lattice values *versus* the Al content from combined XRD/NPD refinement. The *c*-lattice of the charged phase in A10 (refined Al ~0.06) was measured using the Bruker D8 operando XRD data (**Figure S20**).

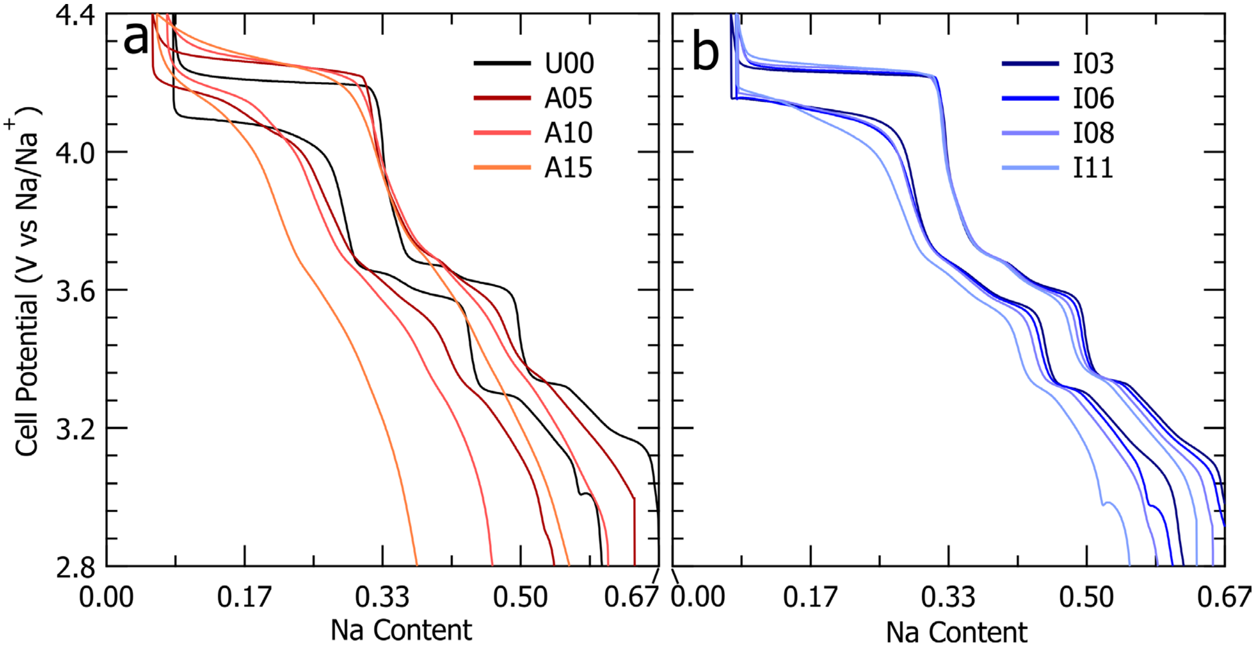

Figure S24. Voltage profiles for (**a**) aliovalent and (**b**) isovalent-substituted NaNMA electrode samples during the first charge-discharge cycle at 0.05 C (8.65 mA g-1).

Table S1. Elemental analysis from inductively coupled plasma optical emission spectroscopy (ICP-OES). Aliovalent samples (**a**) and isovalent samples (**b**). The values were calculated by normalizing the signal against the other elements, *i.e.* $Na_x = I_{Na} / I_{Ni}+I_{Mn}+I_{Al}$ and $Ni_z = I_{Ni} / I_{Ni}+I_{Mn}+I_{Al}$.

a. Name	*Nominal* Al_y	Na_x	Ni_z	Mn_w	Al_y
U00	0.000	0.622	0.334	0.662	0.003
A02	0.025	0.657	0.313	0.663	0.023
A05	0.050	0.658	0.291	0.665	0.044
A07	0.075	0.641	0.264	0.671	0.065
A10	0.100	0.672	0.241	0.669	0.090
A12	0.125	0.666	0.229	0.672	0.099
A15	0.150	0.684	0.195	0.671	0.134

b. Name	*Nominal* Al_y	Na_x	Ni_z	Mn_w	Al_y
I03	1/36 0.027	0.623	0.318	0.654	0.028
I06	2/36 0.055	0.652	0.310	0.642	0.048
I08	3/36 0.083	0.663	0.299	0.628	0.074
I11	4/36 0.111	0.654	0.285	0.617	0.098

Table S2. Values from combined XRD and NPD Rietveld refinement for $Na_{2/3}Ni_{1/3}Mn_{2/3}O_2$. Parameters without standard uncertainty were constrained to these values.

Space Group: *C222₁*, a = 5.01257(5) Å, b = 8.65500(9) Å, c = 11.14642(9) Å, $\alpha = \beta = \gamma = 90°$, Volume = 483.57(2) Å^3, R_{wp} = 8.01%, GoF = 3.11

Site Name	Atom	x	y	z	Occupancy	B (Å^2)	Wyckoff
Na_f	Na	0	0	0	0.215(5)	1.3(2)	4a
Na_f	Na	2/3	0	0	0.215(5)	1.3(2)	4a
Na_e	Na	1/2	1/6	1/2	0.459(6)	5.4(2)	8c
Na_e	Na	5/6	1/6	1/2	0.459(6)	5.4(2)	8c
	Mn	0	0	1/4	0.972(6)	0.343(13)	4b
Mn 1+2	Ni	0	0	1/4	0.034(4)	0.343(13)	4b
	Al	0	0	1/4	0	0.343(13)	4b
	Mn	0	1/3	1/4	0.972(6)	0.343(13)	4b
Mn 1+2	Ni	0	1/3	1/4	0.034(4)	0.343(13)	4b
	Al	0	1/3	1/4	0	0.343(13)	4b
	Mn	0	2/3	1/4	0.057(12)	0.343(13)	4b
Ni 3	Ni	0	2/3	1/4	0.943(7)	0.343(13)	4b
	Al	0	2/3	1/4	0	0.343(13)	4b
O 1	O	0.6778(4)	0.3447(2)	0.3418(5)	1	0.465(14)	8c
O 2	O	0.1436(3)	0.1655(3)	0.3400(5)	1	0.465(14)	8c
O 3	O	0.1785(5)	0.4898(4)	0.3425(6)	1	0.465(14)	8c

Table S3. Values from combined XRD and NPD Rietveld refinement for A02 (nominally $Na_{2/3}[Ni_{1/3-y}Mn_{2/3}Al_y]O_2$ with y = 0.025). Parameters without standard uncertainty were constrained to these values.

Space Group: *C222₁*, *a* = 5.00883(6) Å, *b* = 8.65434(11) Å, *c* = 11.1406(10) Å, *α* = *β* = *γ* = *90* ° Volume = 482.940(10) Å^3, R_{wp} = 7.05%, GoF = 2.68

Site Name	Atom	x	y	z	Occupancy	B (Å^2)	Wyckoff
Na_f	Na	0	0	0	0.213(5)	1.3(2)	4a
Na_f	Na	2/3	0	0	0.213(5)	1.3(2)	4a
Na_e	Na	1/2	1/6	1/2	0.445(5)	4.97(17)	8c
Na_e	Na	5/6	1/6	1/2	0.445(5)	4.97(17)	8c
Mn 1+2	Mn	0	0	1/4	0.968(5)	0.249(12)	4b
	Ni	0	0	1/4	0.032(3)	0.249(12)	4b
	Al	0	0	1/4	0.000(6)	0.249(12)	4b
Mn 1+2	Mn	0	1/3	1/4	0.968(5)	0.249(12)	4b
	Ni	0	1/3	1/4	0.032(3)	0.249(12)	4b
	Al	0	1/3	1/4	0.000(6)	0.249(12)	4b
Ni 3	Mn	0	2/3	1/4	0.064(11)	0.249(12)	4b
	Ni	0	2/3	1/4	0.880(6)	0.249(12)	4b
	Al	0	2/3	1/4	0.056(12)	0.249(12)	4b
O 1	O	0.6783(5)	0.3431(3)	0.3419(6)	1	0.452(13)	8c
O 2	O	0.1435(4)	0.1660(3)	0.3412(6)	1	0.452(13)	8c
O 3	O	0.1782(6)	0.4909(5)	0.3414(7)	1	0.452(13)	8c

Table S4. Values from combined XRD and NPD Rietveld refinement for A05 (nominally $Na_{2/3}[Ni_{1/3-y}Mn_{2/3}Al_y]O_2$ with y = 0.05). Parameters without standard uncertainty were constrained to these values.

Space Group: *C222₁*, *a* = 5.00517(13) Å, *b* = 8.65464(19) Å, *c* = 11.14602(11) Å, $\alpha = \beta = \gamma = 90$ °, Volume = 482.823(18) Å^3, R_{wp} = 6.97%, GoF = 2.74

Site Name	Atom	x	y	z	Occupancy	B (Å^2)	Wyckoff
Na_f	Na	0	0	0	0.221(5)	1.6(2)	4a
Na_f	Na	2/3	0	0	0.221(5)	1.6(2)	4a
Na_e	Na	1/2	1/6	1/2	0.448(6)	5.29(18)	8c
Na_e	Na	5/6	1/6	1/2	0.448(6)	5.29(18)	8c
	Mn	0	0	1/4	0.911(2)	0.282(14)	4b
Mn 1+2	Ni	0	0	1/4	0.0433(19)	0.282(14)	4b
	Al	0	0	1/4	0.001(6)	0.282(14)	4b
	Mn	0	1/3	1/4	0.911(2)	0.282(14)	4b
Mn 1+2	Ni	0	1/3	1/4	0.0433(19)	0.282(14)	4b
	Al	0	1/3	1/4	0.001(6)	0.282(14)	4b
	Mn	0	2/3	1/4	0.108(2)	0.282(14)	4b
Ni 3	Ni	0	2/3	1/4	0.773(7)	0.282(14)	4b
	Al	0	2/3	1/4	0.119(7)	0.282(14)	4b
O 1	O	0.6792(8)	0.3409(5)	0.3423(8)	1	0.534(16)	8c
O 2	O	0.1413(6)	0.1660(6)	0.3422(8)	1	0.534(16)	8c
O 3	O	0.1795(9)	0.4930(7)	0.3394(6)	1	0.534(16)	8c

Table S5. Values from combined XRD and NPD Rietveld refinement for A07 (nominally $Na_{2/3}[Ni_{1/3-y}Mn_{2/3}Al_y]O_2$ with y = 0.075). Parameters without standard uncertainty were constrained to these values.

Space Group: *C222₁*, *a* = 4.99830(3) Å, *b* = 8.65731(5) Å, *c* = 11.14930(12) Å, $\alpha = \beta = \gamma = 90$ °, Volume = 482.450(7) Å^3, R_{wp} = 6.58%, GoF = 2.58

Site Name	Atom	x	y	z	Occupancy	B (Å^2)	Wyckoff
Na_f	Na	0	0	0	0.224(5)	1.6(2)	4a
Na_f	Na	2/3	0	0	0.224(5)	1.6(2)	4a
Na_e	Na	1/2	1/6	1/2	0.437(6)	5.26(18)	8c
Na_e	Na	5/6	1/6	1/2	0.437(6)	5.26(18)	8c
	Mn	0	0	1/4	0.953(18)	0.230(14)	4b
Mn 1+2	Ni	0	0	1/4	0.047(7)	0.230(14)	4b
	Al	0	0	1/4	0.000(19)	0.230(14)	4b
	Mn	0	1/3	1/4	0.953(18)	0.230(14)	4b
Mn 1+2	Ni	0	1/3	1/4	0.047(7)	0.230(14)	4b
	Al	0	1/3	1/4	0.000(19)	0.230(14)	4b
	Mn	0	2/3	1/4	0.09(3)	0.230(14)	4b
Ni 3	Ni	0	2/3	1/4	0.691(16)	0.230(14)	4b
	Al	0	2/3	1/4	0.22(4)	0.230(14)	4b
O 1	O	0.6774(12)	0.3418(8)	0.3426(7)	1	0.528(16)	8c
O 2	O	0.1448(11)	0.1662(8)	0.3410(9)	1	0.528(16)	8c
O 3	O	0.1779(16)	0.4921(11)	0.3403(9)	1	0.528(16)	8c

Table S6. Values from combined XRD and NPD Rietveld refinement for A10 (nominally $Na_{2/3}[Ni_{1/3-y}Mn_{2/3}Al_y]O_2$ with y = 0.10). Parameters without standard uncertainty were constrained to these values.

Space Group: *C222₁*, *a* = 4.99606(3) Å, *b* = 8.65344(5) Å, *c* = 11.15373(8) Å, $\alpha = \beta = \gamma = 90$ °, Volume = 482.210 (6) $Å^3$, R_{wp} = 6.08%, GoF = 2.42

Site Name	Atom	x	y	z	Occupancy	B ($Å^2$)	Wyckoff
Na_f	Na	0	0	0	0.213(5)	1.5(2)	4a
Na_f	Na	2/3	0	0	0.213(5)	1.5(2)	4a
Na_e	Na	1/2	1/6	1/2	0.435(6)	5.6(2)	8c
Na_e	Na	5/6	1/6	1/2	0.435(6)	5.6(2)	8c
	Mn	0	0	1/4	0.936(2)	0.217(14)	4b
Mn 1+2	Ni	0	0	1/4	0.0516(2)	0.217(14)	4b
	Al	0	0	1/4	0.0125(2)	0.217(14)	4b
	Mn	0	1/3	1/4	0.936(2)	0.217(14)	4b
Mn 1+2	Ni	0	1/3	1/4	0.0516(2)	0.217(14)	4b
	Al	0	1/3	1/4	0.0125(2)	0.217(14)	4b
	Mn	0	2/3	1/4	0.11(2)	0.217(14)	4b
Ni 3	Ni	0	2/3	1/4	0.623(7)	0.217(14)	4b
	Al	0	2/3	1/4	0.262(7)	0.217(14)	4b
O 1	O	0.6749(13)	0.3424(8)	0.3415(8)	1	0.563(16)	8c
O 2	O	0.1485(12)	0.1669(8)	0.3398(7)	1	0.563(16)	8c
O 3	O	0.1766(18)	0.4907(12)	0.3422(8)	1	0.563(16)	8c

Table S7. Values from combined XRD and NPD Rietveld refinement for A12 (nominally $Na_{2/3}[Ni_{1/3-y}Mn_{2/3}Al_y]O_2$ with y = 0.125). Parameters without standard uncertainty were constrained to these values.

Space Group: *C222₁*, *a* = 4.99288(3) Å, *b* = 8.64793(5) Å, *c* = 11.16123(13) Å, $\alpha = \beta = \gamma = 90$ °, Volume = 481.920(8) Å^3, R_{wp} = 5.66%, GoF = 2.16

Site Name	Atom	x	y	z	Occupancy	B (Å^2)	Wyckoff
Na_f	Na	0	0	0	0.206(4)	1.2(2)	4a
Na_f	Na	2/3	0	0	0.206(4)	1.2(2)	4a
Na_e	Na	1/2	1/6	1/2	0.432(6)	5.4(2)	8c
Na_e	Na	5/6	1/6	1/2	0.432(6)	5.4(2)	8c
	Mn	0	0	1/4	0.931(4)	0.168(14)	4b
Mn 1+2	Ni	0	0	1/4	0.070(2)	0.168(14)	4b
	Al	0	0	1/4	0.001(2)	0.168(14)	4b
	Mn	0	1/3	1/4	0.931(4)	0.168(14)	4b
Mn 1+2	Ni	0	1/3	1/4	0.070(2)	0.168(14)	4b
	Al	0	1/3	1/4	0.001(2)	0.168(14)	4b
	Mn	0	2/3	1/4	0.204(7)	0.168(14)	4b
Ni 3	Ni	0	2/3	1/4	0.553(7)	0.168(14)	4b
	Al	0	2/3	1/4	0.242(7)	0.168(14)	4b
O 1	O	0.6743(14)	0.3436(9)	0.3404(9)	1	0.549(18)	8c
O 2	O	0.1508(13)	0.1663(9)	0.3407(10)	1	0.549(18)	8c
O 3	O	0.1749(19)	0.4901(13)	0.3423(9)	1	0.549(18)	8c

Table S8. Values from combined XRD and NPD Rietveld refinement for A15 (nominally $Na_{2/3}[Ni_{1/3-y}Mn_{2/3}Al_y]O_2$ with y = 0.15). Parameters without standard uncertainty were constrained to these values.

Space Group: *C222₁*, *a* = 4.99304(3) Å, *b* = 8.64817(6) Å, *c* = 11.17002(15) Å, $\alpha = \beta = \gamma = 90$ °, Volume = 482.331(9) Å^3, R_{wp} = 5.69%, GoF = 2.03

Site Name	Atom	x	y	z	Occupancy	B (Å^2)	Wyckoff
Na_f	Na	0	0	0	0.212(5)	2.0(3)	4a
Na_f	Na	2/3	0	0	0.212(5)	2.0(3)	4a
Na_e	Na	1/2	1/6	1/2	0.394(6)	4.8(2)	8c
Na_e	Na	5/6	1/6	1/2	0.394(6)	4.8(2)	8c
	Mn	0	0	1/4	0.928(19)	0.113(15)	4b
Mn 1+2	Ni	0	0	1/4	0.071(8)	0.113(15)	4b
	Al	0	0	1/4	0.00(2)	0.113(15)	4b
	Mn	0	1/3	1/4	0.928(19)	0.113(15)	4b
Mn 1+2	Ni	0	1/3	1/4	0.071(8)	0.113(15)	4b
	Al	0	1/3	1/4	0.00(2)	0.113(15)	4b
	Mn	0	2/3	1/4	0.23(3)	0.113(15)	4b
Ni 3	Ni	0	2/3	1/4	0.466(17)	0.113(15)	4b
	Al	0	2/3	1/4	0.30(4)	0.113(15)	4b
O 1	O	0.6801(14)	0.3395(9)	0.3417(13)	1	0.569(19)	8c
O 2	O	0.1415(10)	0.1661(10)	0.3416(14)	1	0.569(19)	8c
O 3	O	0.1784(18)	0.4945(14)	0.3398(11)	1	0.569(19)	8c

Table S9. Values from combined XRD and NPD Rietveld refinement for I03 (nominally y = 0.027 in $Na_{2/3}[Ni_{1/3-y/2}Mn_{2/3-y/2}Al_y]O_2$. Parameters without standard uncertainty were constrained to these values.

Space Group: *C222₁*, *a* = 5.00186(10) Å, *b* = 8.67722(15) Å, *c* = 11.12690(9) Å, $\alpha = \beta = \gamma = 90$ °, Volume = 482.932(13) Å^3, R_{wp} = 7.81%, GoF = 2.12

Site Name	Atom	x	y	z	Occupancy	B (Å^2)	Wyckoff
Na_f	Na	0	0	0	0.231(4)	2.14(19)	4a
Na_f	Na	2/3	0	0	0.231(4)	2.14(19)	4a
Na_e	Na	1/2	1/6	1/2	0.485(5)	4.36(14)	8c
Na_e	Na	5/6	1/6	1/2	0.485(5)	4.36(14)	8c
	Mn	0	0	1/4	0.953(13)	0.200(11)	4b
Mn 1+2	Ni	0	0	1/4	0.042(6)	0.200(11)	4b
	Al	0	0	1/4	0.004(15)	0.200(11)	4b
	Mn	0	1/3	1/4	0.953(13)	0.200(11)	4b
Mn 1+2	Ni	0	1/3	1/4	0.042(6)	0.200(11)	4b
	Al	0	1/3	1/4	0.004(15)	0.200(11)	4b
	Mn	0	2/3	1/4	0.03(2	0.200(11)	4b
Ni 3	Ni	0	2/3	1/4	0.857(14)	0.200(11)	4b
	Al	0	2/3	1/4	0.11(3)	0.200(11)	4b
O 1	O	0.6765(9)	0.3449(5)	0.3404(10)	1	0.446(17)	8c
O 2	O	0.1466(7)	0.1667(7)	0.3433(8)	1	0.446(17)	8c
O 3	O	0.1768(12)	0.4884(8)	0.3411(13)	1	0.446(17)	8c

Table S10. Values from combined XRD and NPD Rietveld refinement for I06 (nominally y = 0.055 in $Na_{2/3}[Ni_{1/3-y/2}Mn_{2/3-y/2}Al_y]O_2$). Parameters without standard uncertainty were constrained to these values.

Space Group: *C222₁*, *a* = 5.00065(6) Å, *b* = 8.67720(10) Å, *c* = 11.12697(10) Å, $\alpha = \beta = \gamma = 90$ °, Volume = 482.817(9) Å^3, R_{wp} = 6.52%, GoF = 2.65

Site Name	Atom	x	y	z	Occupancy	B (Å^2)	Wyckoff
Na_f	Na	0	0	0	0.222(4)	1.63(19)	4a
Na_f	Na	2/3	0	0	0.222(4)	1.63(19)	4a
Na_e	Na	1/2	1/6	1/2	0.444(5)	4.55(15)	8c
Na_e	Na	5/6	1/6	1/2	0.444(5)	4.55(15)	8c
	Mn	0	0	1/4	0.955(3)	0.179(11)	4b
Mn 1+2	Ni	0	0	1/4	0.041(3)	0.179(11)	4b
	Al	0	0	1/4	0.004(3)	0.179(11)	4b
	Mn	0	1/3	1/4	0.955(3)	0.179(11)	4b
Mn 1+2	Ni	0	1/3	1/4	0.041(3)	0.179(11)	4b
	Al	0	1/3	1/4	0.004(3)	0.179(11)	4b
	Mn	0	2/3	1/4	0.031(13)	0.179(11)	4b
Ni 3	Ni	0	2/3	1/4	0.860(10)	0.179(11)	4b
	Al	0	2/3	1/4	0.109(16)	0.179(11)	4b
O 1	O	0.6756(5)	0.3456(3)	0.3412(5)	1	0.447(12)	8c
O 2	O	0.1485(4)	0.1666(4)	0.3408(5)	1	0.447(12)	8c
O 3	O	0.1759(7)	0.4878(5)	0.3423(6)	1	0.447(12)	8c

Table S11. Values from combined XRD and NPD Rietveld refinement for I08 (nominally y = 0.083 in $Na_{2/3}[Ni_{1/3-y/2}Mn_{2/3-y/2}Al_y]O_2$). Parameters without standard uncertainty were constrained to these values.

Space Group: *C222₁*, *a* = 4.99930(6) Å, *b* = 8.67585(11) Å, *c* = 11.13357(10) Å, $\alpha = \beta = \gamma = 90$ °, Volume = 482.898(9) Å^3, R_{wp} = 6.92%, GoF = 2.68

Site Name	Atom	x	y	z	Occupancy	B (Å^2)	Wyckoff
Na_f	Na	0	0	0	0.218(4)	1.39(19)	4a
Na_f	Na	2/3	0	0	0.218(4)	1.39(19)	4a
Na_e	Na	1/2	1/6	1/2	0.437(5)	4.67(15)	8c
Na_e	Na	5/6	1/6	1/2	0.437(5)	4.67(15)	8c
	Mn	0	0	1/4	0.959(11)	0.163(12)	4b
Mn 1+2	Ni	0	0	1/4	0.040(2)	0.163(12)	4b
	Al	0	0	1/4	0.001(11)	0.163(12)	4b
	Mn	0	1/3	1/4	0.959(11)	0.163(12)	4b
Mn 1+2	Ni	0	1/3	1/4	0.040(2)	0.163(12)	4b
	Al	0	1/3	1/4	0.001(11)	0.163(12)	4b
	Mn	0	2/3	1/4	0.044(2)	0.163(12)	4b
Ni 3	Ni	0	2/3	1/4	0.855(8)	0.163(12)	4b
	Al	0	2/3	1/4	0.101(8)	0.163(12)	4b
O 1	O	0.6759(5)	0.3455(3)	0.3410(4)	1	0.438(12)	8c
O 2	O	0.1477(4)	0.1662(4)	0.3408(4)	1	0.438(12)	8c
O 3	O	0.1764(6)	0.4882(4)	0.3425(5)	1	0.438(12)	8c

Table S12. Values from combined XRD and NPD Rietveld refinement for I11 (nominal composition y = 0.11 in $Na_{2/3}[Ni_{1/3-y/2}Mn_{2/3-y/2}Al_y]O_2$). Parameters without standard uncertainty were constrained to these values.

Space Group: *C222₁*, *a* = 4.99783(9) Å, *b* = 8.67198(14) Å, *c* = 11.14348(10) Å, $\alpha = \beta = \gamma = 90$ °, Volume = 482.970(12) Å^3, R_{wp} = 7.33%, GoF = 2.79

Site Name	Atom	x	y	z	Occupancy	B (Å^2)	Wyckoff
Na_f	Na	0	0	0	0.219(4)	0.97(17)	4a
Na_f	Na	2/3	0	0	0.219(4)	0.97(17)	4a
Na_e	Na	1/2	1/6	1/2	0.426(5)	5.05(17)	8c
Na_e	Na	5/6	1/6	1/2	0.426(5)	5.05(17)	8c
	Mn	0	0	1/4	0.958(8)	0.174(12)	4b
Mn 1+2	Ni	0	0	1/4	0.039(2)	0.174(12)	4b
	Al	0	0	1/4	0.003(2)	0.174(12)	4b
	Mn	0	1/3	1/4	0.958(8)	0.174(12)	4b
Mn 1+2	Ni	0	1/3	1/4	0.039(2)	0.174(12)	4b
	Al	0	1/3	1/4	0.003(2)	0.174(12)	4b
	Mn	0	2/3	1/4	0.003(2)	0.174(12)	4b
Ni 3	Ni	0	2/3	1/4	0.827(8)	0.174(12)	4b
	Al	0	2/3	1/4	0.124(8)	0.174(12)	4b
O 1	O	0.6746(6)	0.3456(3)	0.3413(4)	1	0.394(13)	8c
O 2	O	0.1481(4)	0.1667(4)	0.3403(4)	1	0.394(13)	8c
O 3	O	0.1774(7)	0.4877(5)	0.3426(5)	1	0.394(13)	8c

Table S13. Charge-balanced chemical formulas from combined refinement of neutron and X-ray powder diffraction.

a. Name	*ICP Al_y*	*Chemical Formula from NPD+XRD*
U00	0.003	$Na_{0.681}[Ni_{0.334}Mn_{0.662}]O_2$
A02	0.023	$Na_{0.677}[Ni_{0.314}Mn_{0.665}Al_{0.021}]O_2$
A05	0.044	$Na_{0.689}[Ni_{0.298}Mn_{0.680}Al_{0.022}]O_2$
A07	0.065	$Na_{0.679}[Ni_{0.254}Mn_{0.686}Al_{0.060}]O_2$
A10	0.090	$Na_{0.663}[Ni_{0.249}Mn_{0.693}Al_{0.058}]O_2$
A12	0.099	$Na_{0.658}[Ni_{0.237}Mn_{0.698}Al_{0.065}]O_2$
A15	0.134	$Na_{0.618}[Ni_{0.206}Mn_{0.707}Al_{0.087}]O_2$
I03	0.028	$Na_{0.710}[Ni_{0.310}Mn_{0.653}Al_{0.037}]O_2$
I06	0.048	$Na_{0.676}[Ni_{0.313}Mn_{0.648}Al_{0.039}]O_2$
I08	0.074	$Na_{0.679}[Ni_{0.310}Mn_{0.652}Al_{0.038}]O_2$
I11	0.098	$Na_{0.669}[Ni_{0.305}Mn_{0.662}Al_{0.033}]O_2$

REFERENCES

(1) Lee, D. H.; Xu, J.; Meng, Y. S. An Advanced Cathode for Na-Ion Batteries with High Rate and Excellent Structural Stability. *Phys. Chem. Chem. Phys.* **2013**, *15* (9), 3304–3312. https://doi.org/10.1039/C2CP44467D.

(2) Kubota, K.; Kumakura, S.; Yoda, Y.; Kuroki, K.; Komaba, S. Electrochemistry and Solid-State Chemistry of NaMeO2 (Me = 3d Transition Metals). *Advanced Energy Materials* **2018**, *8* (17), 1703415. https://doi.org/10.1002/aenm.201703415.

(3) Pfeiffer, L. F.; Dillenz, M.; Burgard, N.; Beran, P.; Roscher, D.; Zarrabeitia, M.; Drews, P.; Hervoches, C.; Mikhailova, D.; Omar, A.; Baran, V.; Paul, N.; Sotoudeh, M.; Busch, M.; Wohlfahrt-Mehrens, M.; Groß, A.; Passerini, S.; Axmann, P. From Structure to Electrochemistry: The Influence of Transition Metal Ordering on Na+/Vacancy Orderings in P2-Type NaxMO2 Cathode Materials for Sodium-Ion Batteries. *J. Mater. Chem. A* **2024**, *13* (1), 540–560. https://doi.org/10.1039/D4TA04786A.

(4) Komaba, S.; Yabuuchi, N.; Nakayama, T.; Ogata, A.; Ishikawa, T.; Nakai, I. Study on the Reversible Electrode Reaction of Na1–xNi0.5Mn0.5O2 for a Rechargeable Sodium-Ion Battery. *Inorg. Chem.* **2012**, *51* (11), 6211–6220. https://doi.org/10.1021/ic300357d.

(5) Lu, Z.; Donaberger, R. A.; Dahn, J. R. Superlattice Ordering of Mn, Ni, and Co in Layered Alkali Transition Metal Oxides with P2, P3, and O3 Structures. *Chem. Mater.* **2000**, *12* (12), 3583–3590. https://doi.org/10.1021/cm000359m.

(6) Vasavan, H. N.; Badole, M.; Saxena, S.; Srihari, V.; Das, A. K.; Gami, P.; Dagar, N.; Deswal, S.; Kumar, P.; Poswal, H. K.; Kumar, S. Identification of Optimal Composition with Superior Electrochemical Properties along the Zero Mn3+ Line in Na0.75(Mn-Al-Ni)O2 Pseudo Ternary System. *Journal of Energy Chemistry* **2024**, *96*, 206–216. https://doi.org/10.1016/j.jechem.2024.04.015.
